# Longitudinal variation in fish prey utilization, trophic guilds, and indicator species along a large subtropical river, China

**DOI:** 10.1002/ece3.4577

**Published:** 2018-11-26

**Authors:** Sai Wang, Tuan‐Tuan Wang, Jin‐Peng Tang, Lin Wang, Yang Yang, Hsing‐Juh Lin, Hao‐Yen Chang, Xing‐An Zhou, Xing Li, Ming Wang

**Affiliations:** ^1^ Research Center of Hydrobiology Department of Ecology Jinan University Guangzhou China; ^2^ Research Center of Tropical and Subtropical Aquatic Ecological Engineering Ministry of Education Engineering Jinan University Guangzhou China; ^3^ State Key Laboratory of Organic Geochemistry Guangzhou Institute of Geochemistry Chinese Academy of Sciences Guangzhou China; ^4^ Department of Life Sciences and Innovation and Development Center of Sustainable Agriculture National Chung Hsing University Taichung Taiwan

**Keywords:** East River, fish feeding habit, predator–prey link, prey taxa, stomach content analysis, trophic structure

## Abstract

Due to the heterogeneous distribution of resources along large rivers, understanding prey utilization by basin‐scale fish assemblages remains a challenge, and thus, recognizing regional fish trophic guilds and indicator species is important. We analyzed the stomach contents of 96 fish species along the subtropical East River in China and identified 8 prey items (29 subcategories). Site‐specific differences in fish diet composition (DC) revealed longitudinal shifts in utilized prey taxa, from upstream lotic to downstream semi‐lentic items, and these were characterized by a decrease in the proportions of epilithic diatoms and aquatic insect larvae (Ephemeroptera and Chironomidae) accompanied by an increase in bivalves (*Corbicula* and *Limnoperna*), shrimps and fishes, and organic sediments. The relative prey consumption weighted by fish abundance and biomass indicated that decreasing insect consumption and increasing detritus consumption were two fundamental vectors governing fish‐centered feeding pathways. Seventeen prey‐oriented fish guilds that were clustered based on DC matrix determined the spatial variation in the fish trophic structure. The cumulative presence of (a) upstream guilds reliant on insects and epiphytes, (b) midstream guilds reliant on hydrophytes, molluscs, and nekton, and (c) downstream guilds reliant on detritus, annelids, and plankton resulted in a longitudinal increase in guild richness, but this continuity was interrupted near the industrialized estuary. The most abundant 28 fish species across the guilds were selected as trophic indicator species; their spatial distribution significantly (*p < *0.05) explained >80% of the environmental and prey variables identified. These species signified the availability of predator–prey links in distinct habitats and the key environmental factors supporting these links. With a high contribution (>51%) of exotic species, an increase in detritivores downstream distinguishes the subtropical East River from temperate rivers. Particularly, in the disturbed lower reaches, the dominance of detritivores prevailed over the predicted increase in other feeding groups (e.g., omnivores and carnivores).

## 1 INTRODUCTION

Conceptualized as a series of physical gradients, river systems provide selective pressures that strongly influence aquatic communities (Horwitz, 1978; Peres‐Neto, Bizerril, & Iglesias, 1995; Seegert, Vondruska, Perry, & Dixon, 2013; Sheldon, 1968). From primary producers to top predators, the communities of river trophic networks display consistent patterns along downstream hydrogeomorphologic units, which can be characterized by heterogeneous food web structures (Aarts & Nienhuis, 2003; Ibañez et al., 2009; Logez, Bady, Melcher, & Pont, 2013; Oberdoff, Guégan, & Hugueny, 1995; Schlosser, 1991; Vannote, Minshall, Cummins, Sedell, & Cushing, 1980). As consumers at the high trophic levels, fish assemblages with different feeding types utilize various prey and thereby govern the top‐down energy cascade through predation effects (Elliott et al., 2007; Welcomme, Winemiller, & Cowx, 2006). To guarantee the integrity of river ecosystems, a focus of ecological management is to recognize the longitudinal patterns of prey utilization by fish species and to maintain the functioning of fish trophic guilds (Vander Zanden, Olden, & Gratton, 2006; Welcomme et al., 2006; Zeni & Casatti, 2014).

The river continuum concept (Vannote et al., 1980), dynamic landscape model (Schlosser, 1991), and riverine productivity model (Thorp & Delong, 1994) have been used to predict the downstream geographical distribution of invertebrates, fish, and algae to maximize resource use. However, as these hypotheses emphasize independent biotic assemblages and their responses to the environment, the evidence for longitudinal changes in fish‐centered feeding pathways and the trophic connections within food webs remains limited. In addition, the generalization of empirical predictions about functional group properties (e.g., type, distribution, and composition) from temperate streams to tropical and subtropical rivers (Hoeinghaus, Winemiller, & Birnbaum, 2007; Statzner & Higler, 1985), which support greater taxonomic diversity, such as region‐specific ichthyofauna (Aarts & Nienhuis, 2003; Lasne, Bergerot, Lek, & Laffaille, 2007), prey taxa, (Hoeinghaus, Winemiller, & Birnbaum, 2007; Statzner & Higler, 1985), and sources of organic inputs (Chang et al., 2012; Humphries, Keckeis, & Finlayson, 2014), has been widely debated. Thus, exploring fish prey utilization throughout an entire basin of subtropical large rivers would not only reveal key predator–prey links that can be used to construct food web diagrams but also provide insights into the spatial heterogeneity of fish trophic structures under contrasting climatic and geomorphologic conditions (Aarts & Nienhuis, 2003; Romanuk, Jackson, Post, McCauley, & Martinez, 2006).

Due to downstream nonstationary resource distribution, which is driven by hydrological dynamics, the prey supply for fish varies greatly across space (Hoeinghaus, Winemiller, & Agostinho, 2007; Poff & Allan, 1995). However, traditional feeding classifications (e.g., carnivore, invertivore, herbivore, etc.) based on behavioral–morphologic traits (Aarts & Nienhuis, 2003) leave questions unanswered regarding what prey are being consumed by cross‐sectional fish assemblages and their relative importance (Buchheister & Latour, 2015). In addition, as fish feed on different prey across habitats, especially generalist feeders (e.g., omnivores), the precise definition of taxonomic prey items and the subclassification of feeding groups are challenging. Thus, it is necessary to use stomach content analysis to identify the fish diet composition (DC) and determine prey‐oriented trophic guilds (Eick & Thiel, 2014; Elliott et al., 2007). Combined with information on species composition, biomass, and abundance, quantifying prey consumption by fish assemblages would inform the identification of energy flows throughout food webs and help reveal the roles that fish play in the structure and functioning of river ecosystems (Elliott et al., 2007; Romanuk et al., 2006).

The links between fish trophic guilds, which are a collection of species with similar feeding habits, and their utilized prey represent the fundamental architecture of food webs (Karr, 1987). In addition to the innate sensitivity of fish species to various perturbations, the presence or absence of fish trophic indicator species, which are nutritionally dependent on their prey, also indicate the available prey sources that function as components of these links (Schiemer, 2000; Schlosser, 1991). Although fish, invertebrate, and diatom indexes have been used to evaluate the ecological health of rivers (Barbour, Gerritsen, Snyder, & Stribling, 1999; Flotemersch, Stribling, & Paul, 2006; Karr, 1981), the significance of exploring versatile ecological indicators that synthesize the effects of single‐object assessments has undergone less exploration. Given strong trophic connectivity, the most abundant species in each guild, which occupy critical nodes within a link have the greatest potential to indicate specialized trophic interactions (Aarts, Van Den Brink, & Nienhuis, 2004; Goldstein & Meador, 2004). Thus, the spatial distribution and migration of fish trophic indicator species associated with particular prey availability along a river would reflect the influence of environmental stressors on the food web structure (Fausch, Torgersen, Baxter, & Li, 2002).

The goal of this study was to uncover the longitudinal patterns in prey utilization by fish along the subtropical East River, an important water source for the Pearl River Delta. Given the great differences in fish fauna among regions and reaches, we hypothesize that fish trophic structures in subtropical rivers are functionally different from their counterparts in temperate streams. Specifically, we aimed to demonstrate that (a) there are longitudinal shifts in prey utilization by fish species and the feeding pathways of fish assemblages, (b) guild classification based on utilized prey taxa by fishes could reveal crucial information that traditional feeding classifications have failed to bring to light, and (c) fish species that represent key trophic links could be used to indicate changes in both environmental factors and prey distribution. To test our hypotheses, experiments were designed to address the following four objectives: (a) to reveal the downstream shifts in prey utilization through basin‐scale fish stomach content analysis, (b) to cluster prey‐oriented fish guilds based on the DC of individual species, (c) to analyze the longitudinal variation in the composition and structure of fish trophic guilds, (d) to quantify the consumption of prey by site‐specific fish assemblages, and (e) to explore the use of the dominant fish species in each guild as indicator species of predator–prey links and environmental factors. Compared with concepts and models based on temperate and tropical rivers, our results provide novel insights into the trophic dynamics of aquatic food webs and ecological assessments of large river systems in the subtropics.

## 2 MATERIALS AND METHODS

### 2.1 Study region and sampling sites

Located in a subtropical monsoon climate, the East River, which is the fourth largest river in China, is one of three main tributaries to the Pearl River system. It is 562 km long and has a drainage area of 35,340 km^2^, and it receives an average of 1,750 mm of precipitation and discharges 32.4 billion m^3^ of water annually. The water resources in the East River are of great importance for flood control, power generation, irrigation, navigation, and water supply in Guangdong Province; and thus, the health of this ecosystem is of the utmost importance to the sustainable development of the Pearl River Delta. However, due to rapid economic development over the past few decades, the river basin has been subject to intense anthropogenic disturbances that have severely impacted the original hydrodynamic and habitat conditions (Lee, Wang, Thoe, & Cheng, 2007).

**Figure 1:**
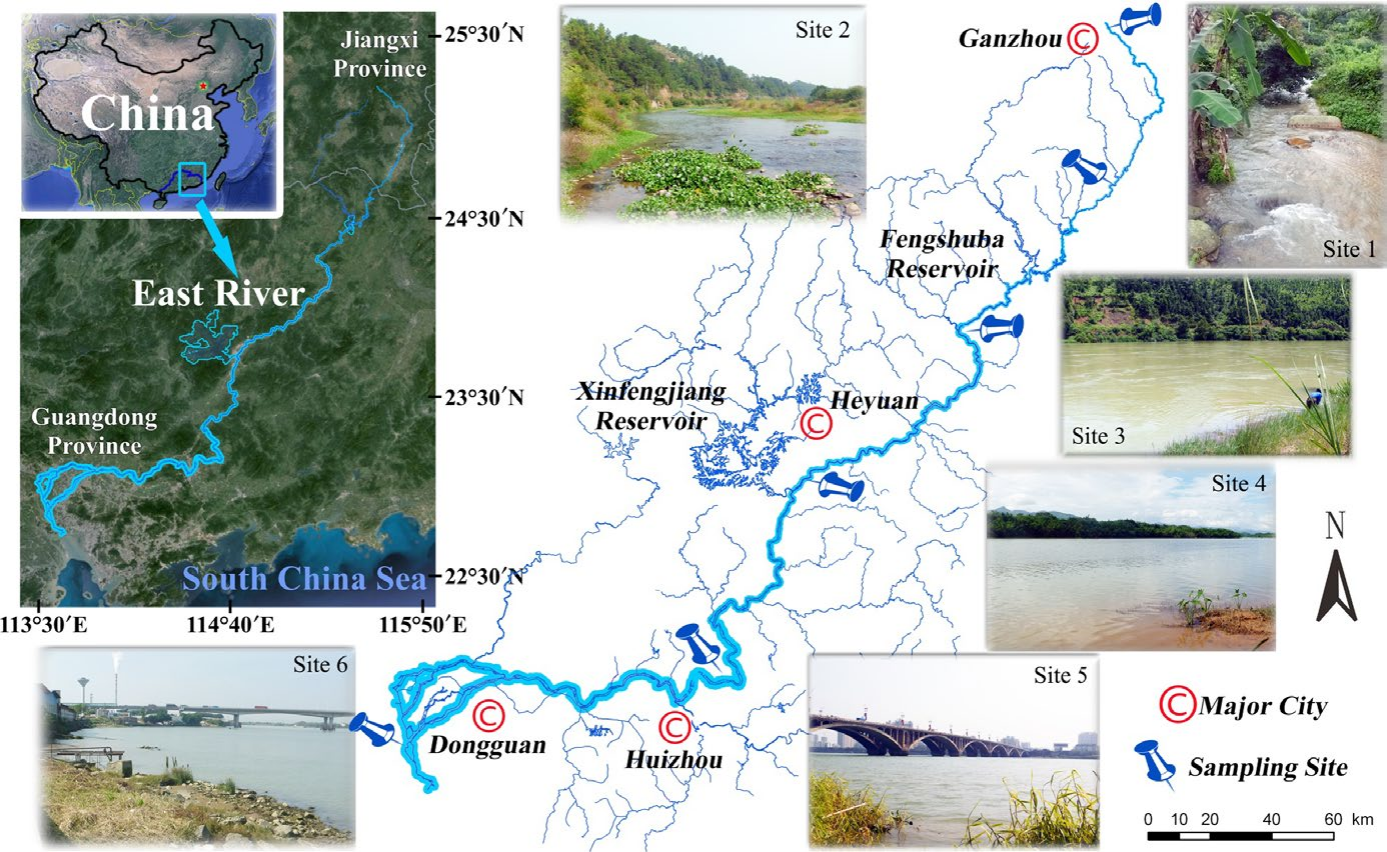
Location of the six sampling sites along the main channel of the East River

Six sampling sites were chosen along the main channel of the East River (Figure 1), and the major habitat characteristics (Table 1) and water parameters were defined (Supporting information Table S1). Wadeable upstream sites 1–2 were in pristine upland areas with boulder and cobble substrates. Site 1 was in the headwaters of mountain stream and bordered by eucalyptus forest on the hillside, and the riparian vegetation was dominated by emergent and littoral annual herbage. At site 2, greater habitat and plant diversity was observed because of the wider channel and floodplain areas, and the dominant submerged plants were *Hydrilla verticillata* and littoral terrestrial macrophytes. Non‐wadeable sites 3–4, which contained a sand substrate, were located in the midstream foothills. Under the influence of Fengshuba Dam upstream, flow regimes were regulated, and seasonal flood pulses were restrained, leading to a slower water velocity and greater depth with fewer floodplains. Typical plants were submerged *Myriophyllum verticillatum* in shallow waters, and extensive bamboo forests were present in the littoral zones. Habitats in the lower reaches have been largely affected by artificial engineering, especially modified tributaries and reinforced banks, resulting in the loss of riffles and floodplains. At urban site 5, submerged plants have been limited by steep revetments and the low transparency of eutrophic waters, and at estuarine site 6 surrounded by industrial zones, few plants were found except for the invasive floating *Eichhornia crassipes*.

**Table 1:** General habitat characteristics of the six sampling sites, averaged for the rainy and dry seasons, along the East River

Parameters/sites	Site 1		Site 2			Site 3			Site 4			Site 5		Site 6		
	Rainy	Dry	Rainy		Dry	Rainy		Dry	Rainy		Dry	Rainy	Dry	Rainy		Dry
Latitude	25°09′02.54″		24°40′25.81″			24°20′34.81″			23°53′05.76″			23°5′53.82″		23°08′01.07″		
Longitude	115°36′25.48″		115°34′53.31″			115°19′22.82″			114°57′51.47″			114°25′8.41″		113°44′43.60″		
Elevation (m)	400.6		288.5			102.3			52.4			21.4		1.8		
River width (m)	12.9	7.3	32.5		20.9	89		73	192		175	447		519		
Discharge (m^3^/s)	1.32	0.44	8.05		2.26	55.2		33.1	109		77.6	326	352	597		568
Riffles (%)	87	95	53		64	15		22	10		15	–		–		
Pools (%)	13	5	30		23	25		20	18		10	–		–		
Canopy cover (%)	95	88	86		73	70		62	100		89	23	16	0		0
Substrate type	Boulders/Cobbles/Coarse gravel					Gravel/Coarse sand						Sand/Fluid mud				
River basin	Headwaters ‐> Upper stream ‐> Middle river ‐> Lower river ‐> Estuary															
Land use pattern	Mountains ‐> Uplands ‐> Agricultural lowland areas ‐> Urban plain ‐> Industrial delta															
Land cover	Mountainous and upland forest ‐> Agricultural crops and lowland shrubs ‐> Residences ‐> Factories/Docks															
Vegetation type	Upland eucalyptus and bamboo forest; riparian arista and herbage; submerged tape grass						Riparian bamboo, arista, reeds, herbage; floating and submerged watermilfoil					Riparian reeds and herbage			–	
Pollution sources	–			–			Nonpoint			–		Point			Point	
Dams	–			–			Upstream			–		Upstream			–	

Notes

The channel width was limited by a bank revetment at sites 5 and 6 and was not influenced by precipitation during the different seasons; absence (–); discharge (m^3^/s) was calculated according to the formula *Q* = *L* × *H* × *V*, where *Q* = flow, *L* = length, *A* = area, and *V* = velocity; flood plain (%) was calculated as a percent of the width of the river channel; substrates were categorized as clay and silt (≤63 μm), sand (63 μm to 2 mm), gravel (2–63 mm), cobbles (63–200 mm), and boulders (≥200 mm).

### 2.2 Fish sampling

Fish were collected from the headwaters to the estuary (sites 1–6 in Figure 1) during the rainy (May and August) and dry (November and January) seasons in 2014–2015; each site was sampled four times over a year following basic guidelines (Barbour et al., 1999; Hauer & Lamberti, 2007). Electrofishing equipment consisted of a 24‐kW generator, a 12V‐160A lithium battery, a silicon‐controlled inverter, and two continuously adjustable voltage and frequency regulators. A copper probe cathode and a 20‐cm‐diameter ring anode with 3‐mm‐mesh net were installed on two 1‐ to 4‐m‐long telescopic insulated rods, respectively. This equipment was used to effectively stun and collect fish (individual weight <10 kg) in a 2‐m‐wide × 2‐m‐long × 3.5‐m‐deep water column. Due to varying water levels, two electrofishing operations were conducted as follows: (a) At wadeable sites 1–2, single‐pass backpack electrofishing was performed simultaneously by two operators moving in zigzag fashion. Electrofishing equipment was adjusted at low voltage and mixed frequency, and the walking speed was controlled to ensure a sampling effort of ~8 m^2^/min over 30 min; (b) At non‐wadeable sites 3–6, a 6‐m‐long welded diesel powered hull boat was used for boat electrofishing, and a bamboo quant was used to propel the boat to eliminate noise disturbance to fish. Electrofishing equipment was adjusted at a high voltage and main frequency, and the paddling speed was controlled to ensure a sampling effort of ~6 m^2^/min. Due to the high water depth, a large scoop net (60‐cm‐diameter, 12‐mm‐mesh) was used by a sternward auxiliary to collect the stunned benthic fish that floated slowly upward. Boat electrofishing was conducted over a distance of 500 m, spanning both river banks at a depth of 1–3 m (Flotemersch et al., 2006). All fish sampling was conducted during daylight hours across diverse habitats. Fish abundance and biomass per unit area were calculated as the number of individuals and the weight mass of the sampled fish specimens divided by the effective sampling area (i.e., electrofishing efforts ×sampling time), which were expressed in inds./m^2^ and g/m^2^, respectively.

### 2.3 Stomach and gut content analysis

To reflect the prey utilization of fish throughout most of their life span, we selected the stomachs or guts of fish specimen close to adult size, including the following: (a) adult individuals of small rheophilic fish species at sites 1–2, and (b) >1‐year‐old individuals from large fish species that reach sexual maturity over several years at sites 3–6. Fish juveniles were collected, counted, weighted, and then released back to the water. The stomachs and guts of individual fish were removed and stored at −18°C. For species that solely utilize bulky prey (e.g., nekton, molluscs, hydrophytes), all stomach contents were removed, identified, and assessed using the gravimetric method (Hyslop, 1980). For species that solely utilize miniature prey (e.g., epiphytes, phytoplankton, detritus), 10 samples of predigested contents from the foreguts were selected, identified, and assessed using the volumetric method (Hellawell & Abel, 1971). For species that utilize both bulky and miniature prey (e.g., insect and epiphyte, molluscs and detritus), the identifiable bulky prey and their fragments were separated from a colloidal mixture composed of gastric juice, miniature prey, and tiny plant or animal debris under a stereo microscope, using a combination of gravimetric and volumetric methods.

The gravimetric method, that is, the direct measurement of the wet mass of prey, was first used to determine the gravimetric proportion of each identified bulky prey compared with the unidentified mixture. The remaining mixture was spread onto a glass plate with a 2‐cm‐wide × 2‐cm‐long × 0.1‐mm‐high groove (uniformly divided across 10 × 10 cells) and diluted with 3–5 times the volume of the mixture in distilled water. The volumetric method, that is, the identification of miniature prey and estimation of their volumes (mm^3^) under an optical microscope, was then used to determine the volumetric proportion of each prey in the mixture, using five mixture samples and 20 cells from each sample randomly selected. Volume calculations were performed as follows: (a) organic particles, algae, protozoans, rotifers, and microcrustaceans were estimated through approximate geometry; (b) the amorphous residue of macroinvertebrates or plants that compacted to fill the plate was estimated from the areas of the cells it covered (Baker, Buckland, & Sheaves, 2014). To unify the dimensions of the DC results, the percentage of each prey item in a given diet was calculated as the relative contribution to the composition of stomach or gut contents:

(1) $$GP_{i}\left(\%\right)=\frac{W_{i\;}}{W_{\text{total}}}\times 100$$

(2) $$VP_{j}\left(\%\right)=\frac{V_{j\;}}{V_{\text{total}}}\times 100$$

(3) $$GP_{j}\left(\%\right)=\frac{W_{\text{mixture}\;}}{W_{\text{total}}}\times \frac{V_{j\;}}{V_{\text{mixture}}}\;\times \;100$$

where GP*_i_* and GP*_j_* are the gravimetric percentages (%) of the bulky prey *i* and the miniature prey *j* in diets; *W_i_*,* W*_mixture_, and *W*_total_ are the wet mass of the bulky prey *i*, the remaining mixture, and the total stomach contents, respectively; *VP_j_* is the volumetric percentages (%) of prey *j* in diets; and *V_j_*,* V*_mixture_, and *V*_total_ are the volumes (mm^3^) of the miniature prey *j*, the mixture sample, and the sample from the predigested contents of the foregut, respectively. Equations ((1)), ((2)), and ((1)) and ((3)) were used for the gravimetric method, volumetric method, and a combination of both methods, respectively.

### 2.4 Prey taxon and diet composition

All the stomach or gut specimen with plumpness <20% were omitted, leaving 2,287 effective specimen detected and analyzed (Supporting information Table S2). As fish species exhibited multiple feeding strategies, prey taxa were aggregated into eight main categories and 29 subcategories (Table 2) to (a) summarize the available prey in site‐specific habitats (e.g., epilithic diatoms in riffles, phytoplankton in slow‐flowing deep waters), (b) identify different fish feeding habits and types (e.g., carnivores swallowing the nekton whole, epiphytivores scraping diatoms, phytoplanktivores filtering algae), (c) define fish trophic guilds with the smallest prey code while preserving major dietary difference, and 4) simplify the presentation of longitudinal variation in prey utilization by identifying representative items. When possible, prey taxa were defined at the family level; however, particular important prey that represented a substantial portion of the DC were retained at the species or genus level (e.g., the hydrophyte *Hydrilla verticillata*, the shrimp *Macrobrachium nipponense*, and the gastropod *Radix*).

**Table 2:**
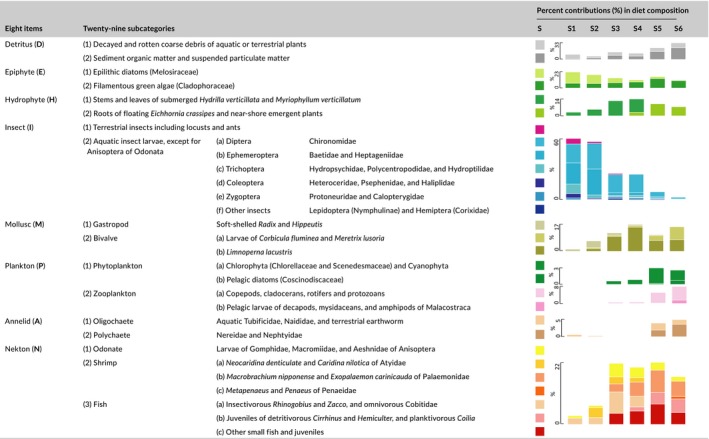
Longitudinal shifts in the utilization of 29 subcategories of 8 main prey items by site‐specific fish assemblages along the East River

The proportion of each prey item in the DC of individual species was summarized gravimetrically or volumetrically using a cluster sampling estimator (Buchheister & Latour, 2015). This estimator accounts for the lack of independence among fish that typically have relatively similar diets and are thus considered pseudoreplicates collected at the same site; it also provides a more accurate population‐level description of the diet than a simple mean because the estimate is weighted by the number of fishes caught at each site. Given our focus on the longitudinal variation in fish prey utilization and trophic structures, the DC of each fish species, which was pooled across months and reaches, was calculated as follows:

(4) $$DC_{\mathrm{ij}}\left(\%\right)=\sum_{k\;=\;1}^{6}\left(\frac{\sum_{h\;=\;1}^{m_{k}}GP_{\mathrm{ih}}\;\text{or}\;VP_{\mathrm{ih}}}{m_{k}}\times \frac{n_{k}}{N}\right)\times 100$$

where DC*_ij_* is the percentage of prey *i* (1–8) in the DC of a given species *j*; GP*_ih_*or VP*_i h_* is the gravimetric or volumetric proportion (%) of prey *i* in the diets of specimen *h* of species *j*;* m_k_* is the number of effective stomach and gut specimens of species *j* sampled at site *k* (1–6); *n_k_* is the number of species *j* sampled at site *k*; and *N* is the total number of individuals of species *j* sampled at sites 1–6.

To determine the longitudinal shifts in prey utilization by fish assemblages (Table 2) at each sampling site, the relative proportions of 29 prey items within the DC were first averaged for individual species and then averaged by combining all species. To quantify the prey consumption by fish assemblage at each site, the proportions of the eight main prey items in the DC were weighted by the contribution of each species to the composition of the assemblage in terms of abundance and biomass:

(5) $$<![CDATA\left[P_{\mathrm{ik}}\left(\%\right)=\sum_{j=1}^{s_{k}}DC_{\mathrm{ij}}\times \left(\frac{n_{j}}{n_{k}}\text{or}\frac{b_{j}}{b_{k}}\right)\times 100\right]]>$$

where *P_i k_* (%) is the percent contribution of prey *i* (1–8) to the total prey consumption by the fish assemblages at site *k*;* s_k_*,* n_j_* (or *b_j_*), and *n_k_* (or *b_k_*) are the number of species, the number of individuals (or biomass) of the given species *j*, and the total number of individuals (or biomass) sampled at site *k*, respectively.

### 2.5 Data analysis

Hierarchical agglomerative clustering of guild‐average linkage was used to identify the trophic guilds of fishes within the study region. The cluster analysis relied on Bray‐Curtis dissimilarities and sequentially aggregated fish species together based on dietary similarity. Statistically significant cluster groupings were identified using a bootstrap randomization technique in which the nonzero values in the DC matrix were resampled and used to generate pseudovalues of Bray‐Curtis dissimilarities under the null hypothesis that no structure existed in the diet matrix (Legendre & Legendre, 2012). A frequency distribution of pseudovalues was generated from 1,000 randomizations of the diet matrix, and the 95th percentile was used as the critical value to determine significance in the cluster analysis of the observed data. According to cluster analysis results, each trophic guild was defined by summing the prey items accounting for the greatest percentages in DC until they reached at least 50% of the total. To designate a guild, codes for the prey taxa were ordered according to the percentages of items in the DC. For example, (a) in guilds I and E, the average percentages of insects and epiphytes in the DC were 83.4% and 74.7%, respectively; and (b) in guild A‐N‐M, the percentages of annelids, nekton, molluscs, and the other five items in the DC were 22.8%, 22.4%, 21.3%, and 33.5%, respectively.

Nonmetric multidimensional scaling (NMDS) was used to corroborate and visualize environment–site and guild–site relationships. NMDS is a nonparametric ordination technique that relies on the rank order of pairwise predator dietary dissimilarities (Bray‐Curtis dissimilarities in this study) and does not make any underlying distributional assumptions of the data (Borcard, Gillet, & Legendre, 2011). NMDS was chosen over other parametric ordination approaches because the environment, diet, and guild data were skewed and not normally distributed. Sampling sites were plotted in ordination space with distance among points positively related to dissimilarity of environmental factors or trophic guild distribution (i.e., sites with similar environments and diets were plotted closer to one another).

The most abundant fish species in each trophic guild were selected to indicate the key predator–prey links along the river. To evaluate the extent to which these predators indicated environmental factors and prey availability (see the distributions of major prey taxa in Table S3), a correspondence analysis was performed to determine the degree of explanation of fish indicator species on environmental and prey variables. The predator–environment and predator–prey relationships were first determined using a detrended correspondence analysis (DCA). A DCA1 gradient length >3.0 (4.08 for predator–environment and 3.87 for predator–prey) indicated a unimodal response; thus, a canonical correspondence analysis (CCA) was applied. For efficiency, stepwise forward selection was used to reduce the number of linearly correlated explanatory variables with axes in the CCA. The statistical significance of the axes derived from each analysis was tested with the maximum number of samples using the Monte Carlo test (999 permutations; Legendre & Legendre, 2012). All multivariate analyses were conducted with R.

## 3 RESULTS

### 3.1 Longitudinal shifts in prey utilization by fish

Combined with the site‐specific habitat conditions (Table 1) and the proportions of the 29 subdivided prey items in the DC of this study (Table 2), fish prey utilization along the East River exhibited longitudinal shifts from upstream lotic taxa in riffles with cobble substrate to downstream lentic taxa in deep waters with silt substrate. The relative ratios of the two subcategories of detritus and epiphytic items indicated that upstream coarse plant debris and epilithic diatoms were replaced by downstream organic sediments and filamentous chlorophyceae. Of the mollusc and hydrophytic items, upstream scrape‐feeding gastropods and submerged *H. verticillata* were replaced by downstream filter‐feeding bivalves and floating *E*. *crassipes*. Of the nekton items, small Atyidae shrimp, insectivorous *Rhinogobius* and *Zacco*, and omnivorous Cobitidae upstream were replaced by large Anisoptera odonate larvae, Palaemonidae shrimp, the pelagic juveniles of detritivorous *Hemiculter* and *Tilapia*, and plantivorous *Coilia* downstream.

The longitudinal decrease in the utilization of lotic prey was essentially dependent on the disappearance of insects from the analyzed DC, which was characterized by a sharp decrease in terrestrial insects at site 2, aquatic Ephemeroptera larvae at site 3, Chironomidae larvae at site 5, and the absence of all insects (<2.57%) at site 6. In contrast, a longitudinal increase in the utilization of lentic prey was first marked by the midstream presence of the bivalve *Limnoperna lacustris*, the larvae of the odonate Anisoptera and the shrimp *Macrobrachium nipponense* in the DC and then by the downstream presence of zooplankton, annelids, and organic sediments. Notably, the disappearance of Chironomidae larvae with the emergence of polychaetes and malacostracans in the DC represented a turning point at site 5 and indicated the final replacement of lotic taxa by lentic taxa as utilized prey.

### 3.2 Clustering of prey‐oriented fish trophic guilds

**Figure 2:**
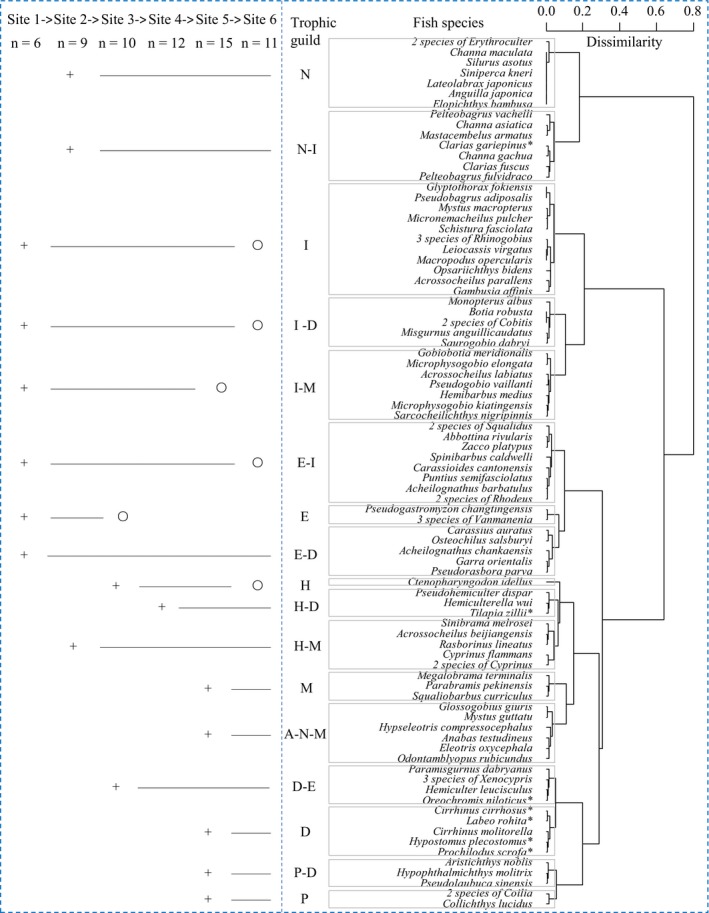
Cluster analysis dendrogram of 17 fish trophic guilds distributed along the East River. The basic data matrix comprises the diet composition of 96 species, including 8 prey items: nekton (N), insect (I), detritus (D), mollusc (M), epiphyte (E), hydrophyte (H), annelid (A), and plankton (P). Each trophic guild is defined by a combination of the codes of the major prey items in diet composition. The number, location of first appearance, continuous emergence and location of eventual loss of each trophic guild are expressed by ‘n’, ‘+’, ‘—’and ‘○’, respectively. Superscript '*' indicates exotic species

Seventeen trophic guilds, each representing collections of species using similar prey, were clustered based on the DC of 96 species (Figure 2), with the richness and distribution of species at sites 1–6 shown in Supporting information Table S2. Along the East River, the richness of species and guilds were lowest in the upstream mountain creek, increased through the midstream agricultural foothills, and reached their highest levels in the lower urban reaches before decreasing sharply near the industrialized estuary. Upstream, 12 fish species were clustered into six trophic guilds, including I, I‐M, I‐D, E, E‐D, and E‐I; at site 2, the number of species and guilds increased to 29 and 9, respectively, due to the emergence of *Cyprinus carpio* in guild H‐M, *Silurus asotus* in guild N, and *Mastacembelus armatus* in guild N‐I.

Midstream, the abdominal suckers *Pseudogastromyzon* and *Vanmanenia* that scrape epilithic diatoms in guild E disappeared at site 3; but *Ctenopharyngodon idellus* in guild H and *Xenocypris argentea* in guild D‐E that graze on hydrophytes and plant debris, respectively, emerged. Interestingly, an increase in the number of guilds at site 4 was caused by two exotic fish species, *Cirrhinus cirrhosus* in guild D and *Tilapia zillii* in guild H‐D. As a result, the number of species and guilds continuously increased from the upper to the middle river, with 37 species clustered into 10 guilds at site 3 and 43 species clustered into 12 guilds at site 4.

In addition to the abovementioned guilds, *Parabramis pekinensis* in guild M, *Mystus guttatu* in guild A‐N‐M, *Hypophthalmichthys molitrix* in guild P‐D, and *Coilia grayii* in guild P emerged downstream. At site 5, 47 fish species were clustered into 15 trophic guilds and the greatest number of species and guilds were recorded, with only guilds E and I‐M missing. However, due to the loss of rheophilic species in guilds containing I, there was a sharp decrease in the number of species and guilds at site 6, with 32 species clustered into 11 guilds.

### 3.3 Composition and structure of fish trophic guilds

**Figure 3:**
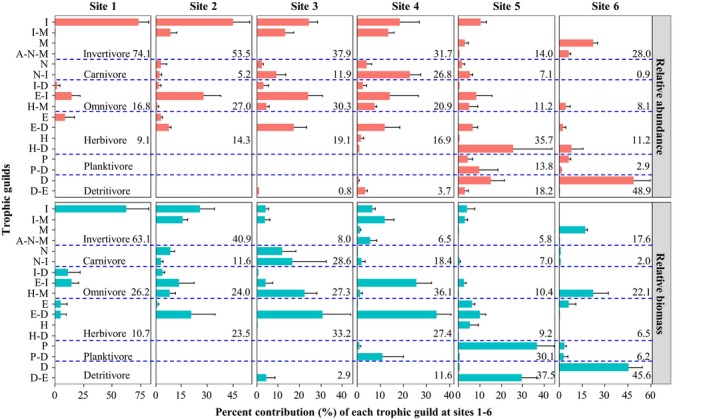
Longitudinal distribution of 17 fish trophic guilds and their percent contributions (%) at the six sampling sites based on relative abundance (number of individuals) and relative biomass. Traditional feeding groups and their composition (%) are listed as invertivores (I, I‐M, M, and A‐N‐M), piscivores (N and N‐I), omnivores (I‐D, E‐I, and H‐M), herbivores (E, E‐D, H, and H‐D), planktivores (P and P‐D) and detritivores (D and D‐E).

As fish species differed greatly in the number and size of individuals identified, two measures of trophic guild composition, abundance and biomass, were calculated separately (Figure 3, see seasonal differences in Figures S1–S2). To facilitate comparison with other studies, the relative proportions of six traditional feeding groups were also determined. Both measures of composition of the six groups showed similar trends in variation: (a) invertivores decreased from site 1 to site 5, with recovery at site 6; (b) carnivores, omnivores, and herbivores increased from site 1 to the midstream sites 3–4 and then decreased until site 6 (an exception was the extremely high abundance of herbivores at site 5 caused by the contribution of the exotic *T*. *zillii*); (c) detritivores emerged at site 3 and continuously increased until site 6; and (d) planktivores emerged at site 5 and then decreased at site 6.

The longitudinal variation in the 17 prey‐oriented trophic guilds showed spatial heterogeneity in fish trophic structure, which was affected by guild composition. In the headwaters and the estuary, the abundance and the biomass composition of fish trophic structure was dominated by guild I (>62%) at site 1 and guild D (>45%) at site 6. However, at sites 2–3, although guilds I and E‐I remained dominant in abundance, their superiority in terms of biomass declined and was replaced by guild E‐D (31%). At sites 4–5, upstream guild I was replaced by guild N‐I (22%) and then by guild H‐D (25%) in terms of highest abundance, whereas upstream guild E‐D was replaced by guild H‐M (34%) and then by guild D (37%) in terms of highest biomass. The proportions of invertivores, carnivores, and planktivores in the fish trophic structure were dependent primarily on the contributions of guilds I, N‐I, and P‐D, respectively. In contrast, the replacement of guild E‐I by H‐M in the internal dominance of omnivores, guild E‐D by H‐D in herbivores, and guild D‐E by D in detritivores indicates the downstream heterogeneous distribution of fish species with specific prey utilization.

### 3.4 Site‐specific prey consumption by fish assemblages

**Figure 4:**
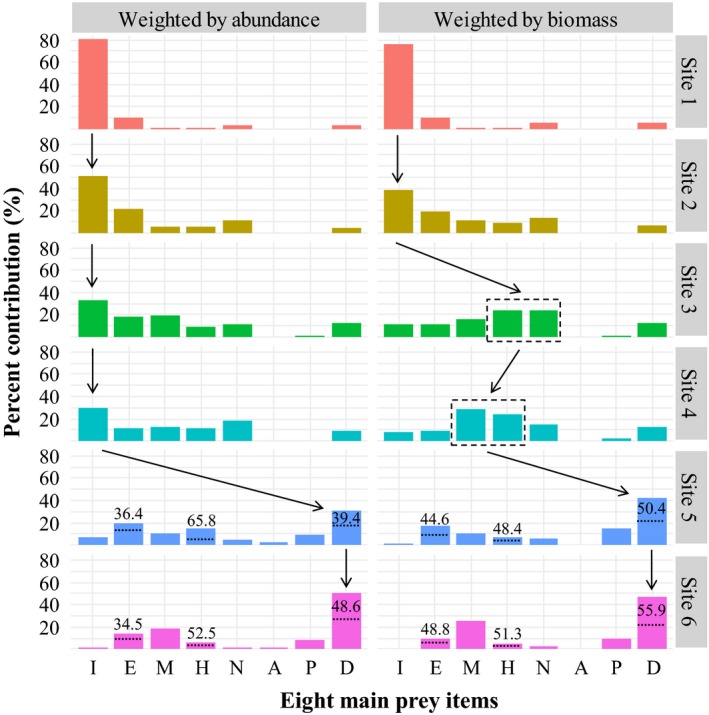
Consumption of 8 prey items by fish assemblages and their percent contributions (%) to the local fish trophic structure at the six sampling sites. N, nekton; I, insect; D, detritus; M, mollusc; E, epiphyte; H, hydrophyte; A, annelid; P, plankton. For each item, the dietary composition of site‐specific fish species was weighted by their relative abundance and relative biomass in the local fish assemblages. At sites 5‐6, the upper portion (%) of each item separated by dotted lines indicates the prey consumed by exotic species

Along the six sampling sites, prey utilization weighted by fish abundance and biomass showed that the downstream decrease in insect consumption and increase in detritus consumption represented the two primary feeding pathways identified in this study (Figure 4). Independent of the composition measure, prey consumption at upstream sites 1–2 was dominated by insects (>41%), while that at downstream sites 5–6 was dominated by detritus (>32%), and both were followed by consumption of epiphytes. Notably, because the high downstream emergence of *T*. *zillii* in guild H‐D and *C*. *cirrhosus* and *Hypostomus plecostomus* in guild D (Table 3), nearly half of the detritus, hydrophyte, and epiphytes was consumed by exotic species at sites 5–6. In the midstream transition areas, as the abundance‐dominant upstream insectivores were gradually replaced by biomass‐dominant midstream omnivores, carnivores, and herbivores (Figure 3), the local prey consumption by fish differed greatly in the measure of assemblage composition. In terms of abundance, the utilization of insects was still highest at sites 3–4, followed by epiphytes at site 3 and nekton at site 4; in terms of biomass, the utilization of hydrophytes and nektons was greatest at site 3, and these groups were replaced by molluscs and hydrophytes at site 4.

**Table 3:**
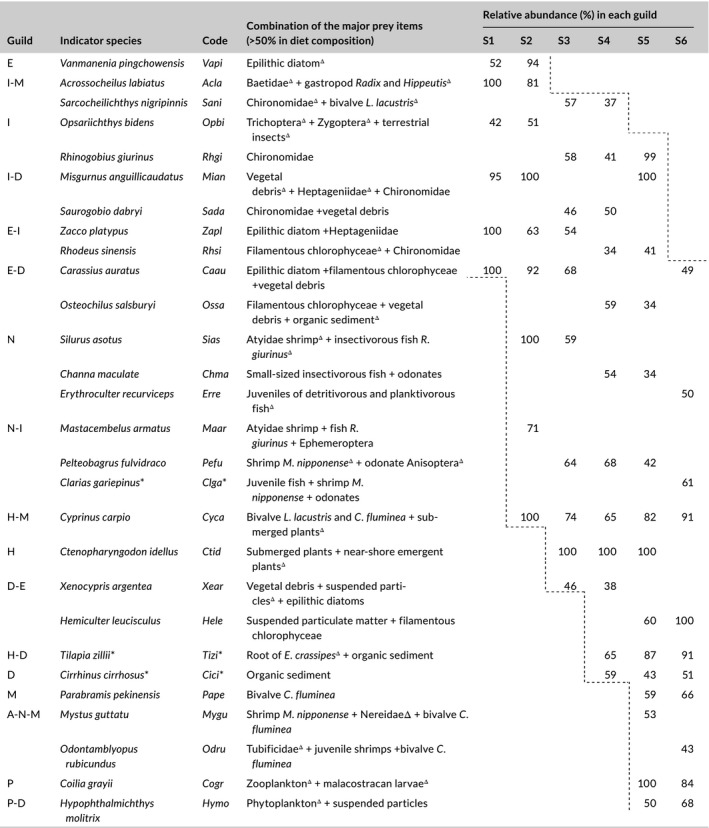
Twenty‐eight fish species selected as trophic indicators of predator‐prey links, their utilized prey items and relative abundance in each guild

### 3.5 Trophic guilds and indicator species in regional zones

**Figure 5:**
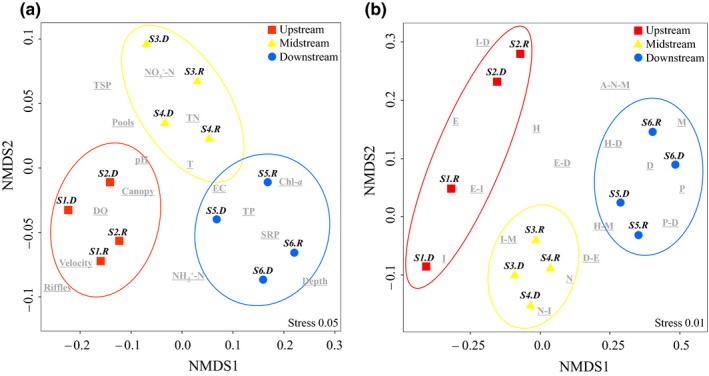
Nonmetric multidimensional scaling (NMDS) ordination of environmental factors and trophic guilds at the six sampling sites. Sampling sites with similar environmental characteristics or fish trophic guilds plot more closely to one another. (a) NMDS plot showing regional zones separated by environments. T, water temperature; DO, dissolved oxygen; EC, electrical conductivity; TN, total nitrogen; NO_X_^−^‐N = NO_3_^−^‐N + NO_2_^−^‐N; TP, total phosphorus; SRP, soluble reactive phosphorus; TSP, total suspended particulates. (b) NMDS plot showing regional zones separated by the abundance distribution (inds./m^2^) of fish trophic guilds. Sampling site during the rainy season (*S.R*) and dry season (*S.D*)

NMDS separated the spatial pattern of the river basin into three statistically significant (*p* < 0.05) regional zones in terms of both environmental factors and the abundance distribution of fish trophic guilds: upstream sites 1–2, midstream sites 3–4, and downstream sites 5–6 (Figure 5). The slight overlap among the midstream and downstream zones reflected the gradual and transitional nature of environmental factors at sites 4–5. As presented, two seemingly independent environmental and trophic gradients emerged from the NMDS plots: (a) a longitudinal decrease in riffle areas, canopy cover, flow velocity, and DO, along with an increase in nitrogen concentrations midstream and in EC, water depth, and phosphorus concentrations downstream (Figure 5a); and (b) a longitudinal decrease in the abundance of guilds containing I and E, with an increase in guilds N and N‐I midstream and in guilds containing D and P downstream (Figure 5b). Basically, the NMDS results indicated that there were significant regional differences along the longitudinal gradient of the river, which was characterized by the environment‐site and guild‐site ordination; thus, we tried to select the fish species that represented the key predator–prey links through their specific prey utilization along this gradient in order to indicate the downstream changes in environmental factors.

The 28 most abundant fish species among the guilds (Table 3) were selected as the indicator species for predator–prey links, which covered all the prey taxa utilized by fish along the river (Figure S3). The longitudinal emergence of these indicator species, for example, the emergence of *Vanmanenia pingchowensis*,* Acrossocheilus labiatus*,* Opsariichthys bidens*, and *C*. *auratus* in the headwaters, illustrates the trophic links based on the utilization of epilithic diatoms, *Radix* gastropods, aquatic insects, and plant debris. Furthermore, the longitudinal replacement of indicator species in the same guild (Table 3) suggests that the trophic guilds could possibly be subclassified to indicate more specialized trophic links. For example, in guild I, *O. bidens*, which utilized Hydropsychidae were replaced by *Rhinogobius giurinus*, which utilized Chironomidae; and in guild I‐M, *A*. *labiatus*, which utilized Baetidae and *Radix* were replaced by *Sarcocheilichthys nigripinnis*, which utilized Chironomidae and *L*. *lacustris*. Notably, the replacements of top predators in guilds N and N‐I were the most obvious: upstream *M. armatus* and *S. asotus*, which utilized Atyidae shrimp and insectivorous fish were replaced midstream by *P*. *fulvidraco* and *Channa maculata*, which utilized odonate larvae and Palaemonidae shrimp and then downstream by *Erythroculter recurviceps*, which utilized small juvenile fish.

### 3.6 Explanation of indicator species on environments and prey

**Figure 6:**
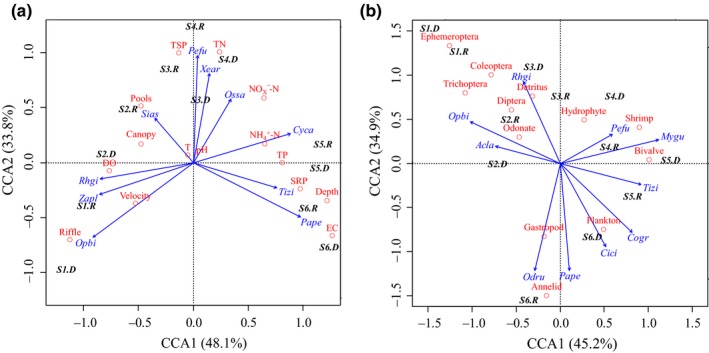
First two axes of the canonical correspondence analysis (CCA) of fish–environment and fish–prey relationships. (a) CCA based on the spatial distribution (inds./m^2^) of fish indicator species and the physicochemical characteristics of habitat and water. T, water temperature; DO, dissolved oxygen; EC, electrical conductivity; TN, total nitrogen; NO_X_^−^‐N = NO_3_^−^‐N + NO_2_^−^‐N; TP, total phosphorus; SRP, soluble reactive phosphorus; TSP, total suspended particulates. (b) CCA based on the spatial distribution of fish indicator species and major prey items. Species abbreviations are summarized in TABLE 3. Sampling site during the rainy season (*S.R*) and dry season (*S.D*)

For the 28 fish selected as trophic indicator species, the CCA results demonstrated significant (*p < *0.05) relationships between their abundance distribution and both environmental factors and prey distribution (Figure 6). Interestingly, for the fish–environment and fish–prey correspondences, forward selection identified 10 indicator species as nonlinear explanatory variables. However, only five of the 10 selected indicator species were common, suggesting that these fish species tended to indicate specific environment or prey variables. The eigenvalues of the two fish‐environment canonical axes were 0.223 and 0.103, which explain 48.1% and 33.8% of the environmental factors, respectively (Figure 6a); the eigenvalues of the two fish‐environment canonical axes were 0.214 and 0.097, which explain 45.2% and 34.9% of the prey distribution factors, respectively (Figure 6b). Only the first two axes represented a significant variation (*p < *0.01) from random components with high correlations of CCA1 = 0.945, 0.933, and CCA2 = 0.882, 0.795. The CCA results demonstrated that fish trophic indicator species explain a high degree of the variation in environments and prey, with *R. giurinus*,* O*. *bidens*,* P. fulvidraco*,* P. pekinensis*, and *T*. *zillii* providing the most explanation.

The upstream indicator species, such as *O*. *bidens*, which utilized terrestrial and aquatic insects, *Zacco platypus*, which utilized epiphytes, and *A*. *labiatus*, which utilized soft‐shelled gastropods, were associated with high water velocity and dissolved oxygen (DO), widespread riffles with cobble substratum, and dense riparian vegetation cover. The midstream indicator species, such as *C. carpio*, which utilized submerged plants, and *P. fulvidraco*, which utilized odonate larvae and shrimps, were associated with increased river depth and channel width and the highest nitrogen concentrations. Notably, *X*. *argentea*,* H*. *leucisculus*, and *Osteochilus salsburyi*, which utilized deposited plant debris and organic particles as well as the emergent *E. recurviceps*, a rare pelagic carnivore, were associated with slower water velocity and increased water surface and depth. Two exotic species, *C*. *cirrhosus* and *T*. *zillii*, which utilized organic sediments and the roots of floating *E. crassipes*, were associated with semi‐lentic eutrophic waters in the downstream urban reaches. In addition, *P. pekinensis*, the filter‐feeding *H. molitrix*, and *M. guttatu* and *Glossogobius giuris*, which utilized the bivalve *C. fluminea*, plankton, and annelids and shrimp, were associated with the highest conductivity, chlorophyll *a*, and phosphorus concentrations near the industrialized estuary.

## 4 DISCUSSION

### 4.1 Longitudinal variation in fish trophic guilds along the East River

Along the East River, both the fish trophic guilds and their prey utilization were selected based on the longitudinal physical gradients. Similar to studies conducted in temperate and tropical streams (Ibanez et al., 2007; Oberdorff, Pont, Hugueny, & Chessel, 2001; Petry & Schulz, 2006; Wolff, Carniatto, & Hahn, 2013), insectivores, especially those in guild I, which feed exclusively on insects, were dominant in riffles of the upper East River where harsh physical conditions (e.g., high velocity and shallow water) are present; however, their abundance and biomass continuously declined until the estuary. Such a longitudinal decrease is likely attributed to the unavailability of essential habitats for rheophilic fish and aquatic insects downstream (Vannote et al., 1980; Wang, Lee, Cheng, & Duan, 2008), such as the lack of flowing waters and rocky substrates that result in weak hydrodynamics (Angermeier & Karr, 1983; Hoeinghaus, Winemiller, & Birnbaum, 2007; Karr, 1987).

Although the predicted downstream increases in omnivores, herbivores, and carnivores (Schlosser, 1991; Vannote et al., 1980) were observed from the upper to the middle East River, this tendency was interrupted by the dominance of guild D, that is, detritivores, and the filter‐feeding guild P‐D, that is, planktivores, in the lower reaches. As reported in tropical rivers (Ibanez et al., 2007; Pouilly, Barrera, & Rosales, 2006), large numbers of detritivores, which benefit from the decomposition of plentiful organic matter under high water temperatures and have long intestines that permit slow digestion (Petry & Schulz, 2006; Wolff et al., 2013), often prevail over the contributions of the other guilds.

The predicated downstream decrease in invertivores (Ibanez et al., 2007; Petry & Schulz, 2006; Pouilly et al., 2006; Vannote et al., 1980) was observed at sites 1–5 (Figure 3) and is primarily explained by the downstream decrease of insectivores in guilds I, I‐M, and I‐N. However, at estuarine site 6, the absence of invertivores in the fish trophic structure, which was caused by the disappearance of insectivores, was addressed by the downstream emergence of molluscivores in guild M in high abundance and biomass. Generally, these results are consistent with the findings of Goldstein and Meador (2004) who noted that the relationship between the distribution of feeding groups and stream size vary with specific fish feeding habits and study areas; our results suggest that a longitudinal increase in detritivores accompanied by a decrease in insectivores is the only pattern that is realistic for the subtropical East River. This pattern distinguishes the trophic structure of fish in tropical/subtropical rivers from their temperate counterparts.

### 4.2 Downstream shifts in prey utilization by fish assemblages

The longitudinal decrease in insect utilization and increase in detritus utilization by site‐specific fish assemblages constitutes two opposing vectors governing the main energy pathways along the East River, with midstream transitions indicated by the high utilization of hydrophytes, molluscs, and nekton. In the headwaters of temperate streams, organic inputs into aquatic food webs depend on allochthonous terrestrial detritus (Chang et al., 2012; Matveev & Robson, 2014; Vannote et al., 1980), but our results support the findings from tropical rivers that autochthonous aquatic insects and epilithic diatoms create basic prey sources for upstream fish (Angermeier & Karr, 1983; Moyle & Senayake, 1984). Such differences might be attributed to the sparse canopy of the open eucalypt forests along the upper East River and the year‐round high temperatures in the subtropics, which stimulate the production of riverine invertebrates and autotrophic producers (Davies, Bunn, & Hamilton, 2008; Mazzoni & Lobón‐Cerviá, 2000).

As bivalves, decapod crustaceans, and odonate larvae have been commonly found in subtropical lowland streams with nearshore submerged plants (Jacobsen, Cressa, Mathooko, & Dudgeon, 2008), their availability in the middle East River might explain the increased proportions of these prey in the DC (Wang et al., 2008). In addition, the increased water depth, slower velocity, and sand/silt substrate constrained guild I, which feeds on aquatic insects, and guild E, which scrape epilithic diatoms. Nevertheless, these conditions were favorable to the survival of larger species living in pools (Petry & Schulz, 2006; Wolff et al., 2013), such as the herbivores in guild E‐D, the omnivores in guild H‐M, and the carnivores in guilds N and N‐I. As a result, midstream prey utilization weighted by fish biomass was notably increased for hydrophytes, molluscs, and nekton, whereas that weighted by abundance continued to be dominated by insects. Consequently, our findings demonstrate that the longitudinal variation in the fish trophic structure was not only associated with downstream shifts in prey utilization and guild distribution but also differed greatly in assemblage composition.

Interestingly, due to the diverse habitats at the intersections of tributaries and the mainstream and availability of plankton downstream, the highest guild richness was predicted in both the intermediate (3–4 order) (Minshall et al., 1985; Tejerina‐Garro et al., 2005; Vannote et al., 1980) and large (>6 order) river sections (Adite & Winemiller, 1997; Elliott et al., 2007; Peres‐Neto et al., 1995), respectively. Indeed, our findings (Table 2) suggest such regional variations were determined by the location where lotic prey in riffles (e.g., aquatic insects and epilithic diatoms) co‐occurred with lentic prey in deep waters (e.g., bivalves, plankton and annelids). Typically, at site 5, the last remaining chironomid larvae with the downstream emergent polychaetes observed in DC marked the utilization of various prey items that finally yielded the highest guild richness.

### 4.3 Unique patterns of the fish trophic structure in the lower reaches

Along the East River, the successive presence of upstream guilds that utilized aquatic insects, epiphytes, and gastropods; midstream guilds that utilized submerged plants, bivalves, odonate larvae, shrimps, and fish; and downstream guilds that utilized organic sediments, plankton, and annelids (Figure 2) accounted for three longitudinal gradients found to govern the fish trophic structure. It could be expected that the cumulative addition of guilds that had already appeared upstream would lead to peak guild richness somewhere downstream (Eick & Thiel, 2014; Vannote et al., 1980). As expected, the greatest richness of fish species and trophic guilds was observed at site 5, supporting the prediction that the complex downstream habitats support diverse ecological types of fish assemblages (Eick & Thiel, 2014; Welcomme et al., 2006). However, although the exotic species with high abundance and biomass in guild D increased guild richness, their heavy reliance on organic sediment resulted in the dominance of detritus in prey consumption by local fish assemblages (Figure 4). Thus, the fish trophic structure at site 5 should be described as having relative integrity of guild richness but becomes unbalanced in prey consumption (Romanuk et al., 2006).

Near the highly industrialized estuary, a sharp decrease in both species and guild richness at site 6 resulted from the loss of rheophilic species in guilds containing I. However, in contrast to findings that degraded fish assemblages in disturbed areas are represented by tolerant omnivores (Karr, 1981; Oberdorff et al., 2001; Schiemer, 2000), our results showed that the fish trophic structure in the lower East River was dominated by detritivores, especially the exotic *C. cirrhosis* and *H. plecostomus* in guild D. Southerland et al. (2007) suggested that environmental stressors (e.g., regional climate and fluvial morphology) in urban and industrial zones eliminates sensitive functional groups and reshapes the original food webs. Accordingly, the patterns observed at site 6, which interrupts the longitudinal connectivity of fish trophic guilds, indicate that accurate predictions of fish trophic structure must incorporate the impacts of anthropogenic disturbance and biological invasion.

### 4.4 Ecological application of fish trophic indicator species

The selected fish trophic indicator species and their prey utilization reflect the basic geomorphologic and physicochemical parameters along the East River (Figure 5). The locations where these indicators appeared, became dominant, and eventually disappeared could be used to judge changes in the fish trophic structure in response to regional environments and prey availability (Welcomme et al., 2006; Zeni & Casatti, 2014). For instance (Figure S3), the upstream links connecting *O. bidens*,* V. pingchowensis* and *A. labiatus* with insects, diatoms, and gastropods indicate high water velocity, widespread riffles, and a cobble substratum. Completely dissimilar from the upstream links, the downstream links connecting *H. molitrix*,* P. pekinensis* and *M. guttatu* with plankton, bivalves, and annelids indicate semi‐lentic eutrophic waters with greater depth and a silt substrate. The impacts of the upstream Fengshuba Dam, which obstructs the hydrodynamic connectivity of the river, stimulated the downstream loss of guild I and the emergence of guild D, which reflects the reallocation of prey sources in riffle habitats (Aarts et al., 2004; Wang et al., 2008).

One problem that has received little attention is the importance of guilds that utilize just one prey item in the maintenance of food‐web frameworks. Along the East River, such single‐prey guilds, which consisted of upstream I and E, midstream N and H, and downstream D, M and P, existed or were dominant only in specific river sections (Figure 3). The basin‐scale distribution of these guilds exhibited obvious spatial heterogeneity, which could possibly be explained by the specialized prey supply under harsh habitat conditions, such as the epilithic diatoms in rapids and the organic sediments and plankton in semi‐lentic waters with silt substrate (Tejerina‐Garro et al., 2005). In addition, the appearance of these single‐prey guilds forecasted the components necessary for other composite guilds (e.g., I‐E, H‐M, and D‐P) and implied the possible combinations of predator‐prey links. Correspondingly, guild richness was highest at site 5, where guilds I, N, H, D, M, and P were observed, and then sharply decreased at site 6, where guilds I and H disappeared. In contrast to site 5, where guilds I and H could be sampled near shore, the absence of these guilds at site 6 was largely affected by the bank revetment that destroyed the original riparian zones (Aarts et al., 2004).

The present synthesis of fish diets and trophic structures in the subtropical East River provides insights into the spatial heterogeneity of the fish trophic structure under contrasting climatic and geomorphologic conditions. Additionally, prey‐oriented fish trophic guilds can aid the development of indicators of ecosystem status, such as the trophic indicator species of key predator–prey links, which have proven to be responsive to changes in ecosystem status and fishing pressure (Aarts & Nienhuis, 2003). Such indicators can operate within a suite of metrics to help establish ecosystem reference points, assess the direct and indirect effects of anthropogenic and environmental perturbations, and control rules or decision criteria to inform management actions (Buchheister & Latour, 2015). More generally, this work contributes to the collective understanding of the structure, function, and ecological gradients of river food webs, which is fundamental to more holistic ecosystem approaches to ecological management.

## CONFLICT OF INTEREST

The authors declare that they have no competing interests.

## AUTHORS’ CONTRIBUTIONS

S.W. conceived the ideas and designed the methodology; S.W., X.‐A.Z., L.W., M.W., X.L., and T.‐T.W. collected and analyzed the data; S.W., Y.Y, H.‐J.L., and H.‐Y.C. led the writing of the manuscript. All authors contributed critically to the drafts and gave final approval for publication.

## DATA ACCESSIBILITY

All data supporting this study are provided as supplementary information accompanying this manuscript.
