# Supplementary material for: Longitudinal variation in fish prey utilization, trophic guilds, and indicator species along a large subtropical river, China
Source: Ecol Evol. 2018 Nov 26;8(23):11467–83. doi: 10.1002/ece3.4577 (PMC6303697; doi:10.1002/ece3.4577)
Supplement: Supplementary file 1 [file ECE3-8-11467-s001.docx]

**SUPPLEMENTARY MATERIAL**

**Method S1** Physical and chemical parameters of water quality

At each site, channel width (m), depth (m), and current velocity (m·s^−1^) were measured using a GPS, portable [propeller](app:ds:propeller) flow meter (LS25-3C, Tongda, China), and depth finder (SM-5A, Speedtech, USA), respectively. Temperature (°C), dissolved oxygen (mg·L^-1^), pH, and electrical conductivity (μS·cm^−1^) were measured using a YSI meter (YSI Pro Plus, Yellow Springs, OH, USA). To determine total suspended matter (TSM) and chlorophyll *a* concentration (Chl-*a*), 2 L and 500 mL of water were filtered through pre-combusted (at 450 °C for 6 h) glass fiber filters (Whatman GF/F, *n* = 6) and cellulose acetate membrane filters (0.45 μm, *n* = 6), respectively. Samples for total nitrogen (TN), total [phosphorus](app:ds:phosphorus) (TP), total oxidized nitrogen (NO_X_-N = NO_3_-N + NO_2_-N), [ammonia](app:ds:ammonia) [nitrogen](app:ds:nitrogen) (NH_4_^+^-N), and soluble reactive phosphorus (SRP) were preserved in acid-washed 300 mL polyethylene bottles (for each *n* = 6). All filters and water samples collected in the field were stored at -18 °C. In the laboratory, TSM filters were dried at 105 °C to constant weight; for pre-treatment steps, Chl-*a* was extracted from homogenized filters using acetone, NO_X_-N, NH_4_^+^-N and SRP samples were filtered through Whatman GF/F, and TN and TP samples were digested with alkaline persulfate at 120 °C. Leaching solutions of Chl-*a*, digested TN and TP, filtered NO_X_-N, NH_4_^+^-N, and SRP were determined using colorimetric methods. All water quality parameters were determined according to standard methods for water and wastewater analysis (SEPA 2002).

**Method S2** Sampling of macroinvertebrates, plant debris, and hydrophytes

All the sampling of macroinvertebrates, plant debris, and hydrophytes were conducted during the rainy (April and July 2016) and dry season (October 2016 and January 2017) from headwaters to the estuary (sites 1-6) following basic guidelines (Barbour et al. 1999; Hauer and Lamberti 2007). Two sampling methods for macroinvertebrates, plant debris, and submerged hydrophytes are as follows: 1) At sites 1-2 in mountain streams with cobble/gravel beds, the substrates were washed and sieved with a kick-net with 0.5 mm-mesh, with sampling areas of 1-5 m^2^. Plant debris and submerged hydrophytes were separated and weighted in the field, with remaining macroinvertebrates collected. 2) At sites 3-6 in the middle and lower reaches with large water depth and sand bed, a Petersen grab bucket sampler was used, with a sampling area of 0.04 m^2^. At each sampling site, 25 grab samples were taken across the river section. The samples at sites 3-6 were treated in the same way as samples at sites 1-2. After sampling, macroinvertebrates were preserved in ethanol and were subsequently identified in the laboratory to species or genus level (Wang et al. 2008).

**Table S1** Physicochemical parameters of water quality (standard deviation in parentheses) at the 6 sampling sites along the East River, averaged for rainy and dry seasons.

| Parameters |  | Site 1 | |  |  | Site 2 | |  |  | | Site 3 | |  |  | Site 4 | |  |  | Site 5 | |  |  | | Site 6 | |  |  |
| --- | --- | --- | --- | --- | --- | --- | --- | --- | --- | --- | --- | --- | --- | --- | --- | --- | --- | --- | --- | --- | --- | --- | --- | --- | --- | --- | --- |
|  | Rainy | | Dry | | Rainy | | Dry | | | Rainy | | Dry | | Rainy | | Dry | | Rainy | | Dry | | | Rainy | | Dry | | |
| T (°C) | 23.9 | | 7.3 | | 24.5 | | 7.8 | | | 25.7 | | 15.8 | | 28.3 | | 14.6 | | 30.5 | | 14.2 | | | 30.8 | | 15.00 | | |
| Depth (m) | 0.27 | | 0.15 | | 0.40 | | 0.22 | | | 2.48 | | 2.04 | | 3.43 | | 2.82 | | 7.28 | | 6.64 | | | 8.82 | | 7.94 | | |
| Velocity (m·s^-1^) | 0.47 | | 0.39 | | 0.61 | | 0.55 | | | 0.24 | | 0.19 | | 0.17 | | 0.15 | | 0.14 | | 0.13 | | | 0.11 | | 0.09 | | |
| pH | 6.25 | | 6.50 | | 6.42 | | 6.61 | | | 6.43 | | 6.53 | | 6.48 | | 6.71 | | 6.77 | | 7.01 | | | 5.30 | | 5.96 | | |
| DO (mg·L^-1^) | 8.37  (1.13) | | 13.70  (2.07) | | 8.24  (1.52) | | 14.93  (2.21) | | | 5.73  (0.51) | | 9.43  (1.28) | | 6.63  (0.96) | | 10.78  (1.77) | | 5.89  (0.69) | | 9.06  (1.60) | | | 2.82  (1.04) | | 8.83  (2.09) | | |
| EC (μS·cm^−1^) | 43.7 | | 35.2 | | 51.9 | | 42.3 | | | 89.9 | | 78.4 | | 86.8 | | 74.8 | | 96.0 | | 77.5 | | | 153.2 | | 121.1 | | |
| TSP (mg·L^-1^) | 8.78  (3.41) | | 23.89 (11.28) | | 10.59 (6.30) | | 15.60 (1.57) | | | 29.53 (3.86) | | 21.18 (1.28) | | 24.67 (3.02) | | 19.78 (1.71) | | 14.19 (1.35) | | 12.98 (1.06) | | | 20.69 (1.77) | | 15.34 (2.30) | | |
| TN (mg·L^-1^) | 1.31  (0.45) | | 0.99 (0.21) | | 1.73  (0.21) | | 1.69 (0.38) | | | 3.36  (0.61) | | 2.88 (0.03) | | 2.74 (0.12) | | 2.35  (0.24) | | 2.89 (0.24) | | 1.92  (0.08) | | | 3.67 (0.18) | | 3.08  (0.23) | | |
| NH_4_^+^-N (mg·L^-1^) | 0.09 (0.06) | | 0.14 (0.05) | | 0.18 (0.07) | | 0.20 (0.08) | | | 0.21 (0.02) | | 0.17 (0.01) | | 0.35 (0.09) | | 0.20  (0.02) | | 0.13  (0.04) | | 0.19 (0.07) | | | 0.46 (0.01) | | 1.03  (0.01) | | |
| NO_X_^-^-N (mg·L^-1^) | 0.90 (0.36) | | 0.65 (0.05) | | 0.95 (0.15) | | 0.85 (0.15) | | | 2.58 (0.17) | | 2.38 (0.05) | | 2.07 (0.04) | | 2.03 (0.03) | | 1.65  (0.03) | | 1.32 (0.04) | | | 1.95 (0.03) | | 1.72 (0.01) | | |
| TP (mg·L^-1^) | 0.035 (0.008) | | 0.012 (0.009) | | 0.061 (0.029) | | 0.037 (0.018) | | | 0.064 (0.005) | | 0.061 (0.002) | | 0.068 (0.010) | | 0.066 (0.008) | | 0.079 (0.032) | | 0.042 (0.005) | | | 0.156 (0.014) | | 0.119 (0.014) | | |
| SRP (mg·L^-1^) | 0.008 (0.004) | | 0.005 (0.004) | | 0.014 (0.012) | | 0.011 (0.006) | | | 0.020 (0.002) | | 0.016 (0.001) | | 0.024 (0.008) | | 0.020 (0.003) | | 0.030 (0.002) | | 0.012 (0.003) | | | 0.059 (0.015) | | 0.053 (0.003) | | |
| Chl-*a* (mg·L^-1^) | 0.74 (0.68) | | 0.65 (0.10) | | 1.17 (0.19) | | 0.93 (0.09) | | | 1.57 (0.14) | | 1.32 (0.55) | | 3.34 (0.96) | | 1.87 (0.61) | | 17.40 (5.43) | | 4.52  (0.05) | | | 9.01 (3.83) | | 2.92 (0.56) | | |

T, water temperature; DO, dissolved oxygen; EC, electrical conductivity; TSP, total suspended particulate; TN, total nitrogen; NO_X_^－^-N, NO_3_^-^-N + NO_2_^-^-N; TP, total phosphorus; SRP, soluble reactive phosphorus.

**Table S2** The relative abundance and the diet composition (DC) of 96 fish species sampled at sites 1-6 along the East River

At each sampling site, the relative abundance (%) was the percent contribution of a species in the total number of fish individuals, averaged for the rainy and dry seasons. For each fish species, N_ind_ is the number of individuals sampled sites 1-6, and N_DC_ is number of effective stomach or gut specimen analysed. Eight main prey items: Detritus (D), Epiphyte (E), Hydrophyte (H), Insect (I), Mollusk (M), Plankton (P), Annelid (A), Nekton (N). Twenty-eight fish trophic indicators of predator-prey links that are selected from each guild are marked in blue. "*" signifies exotic species.

| **Fish species in each trophic guild** | **N_ind_**  5470 | **Relative abundance (%) at each sampling site** | | | | | | **N_DC_**  2287 | **Percent contributions (%) of 8 prey items in DC** | | | | | | | |
| --- | --- | --- | --- | --- | --- | --- | --- | --- | --- | --- | --- | --- | --- | --- | --- | --- |
|  |  | **S1** | **S2** | **S3** | **S4** | **S5** | **S6** |  | **I** | **E** | **M** | **H** | **N** | **A** | **P** | **D** |
| **N** |  |  |  |  |  |  |  |  | Contribution of nekton > 90.1 | | | | | | | |
| *Erythroculter recurviceps* | 16 |  |  | 0.4 | 0.9 | 0.4 | 0.3 | 10 |  | 0.5 | 3.0 | 6.2 | 90.1 |  |  | 0.1 |
| *Erythroculter hypselonotus* | 1 |  |  |  | 0.2 |  |  |  |  |  |  |  |  |  |  |  |
| *Channa maculata* | 27 |  |  | 0.5 | 2.1 | 0.6 | 0.2 | 2 | 0.2 |  |  |  | 99.8 |  |  |  |
| *Silurus asotus* | 73 |  | 3.2 | 1.5 | 0.3 | 0.5 |  | 33 |  |  |  |  | 100 |  |  |  |
| *Siniperca kneri* | 3 |  |  |  | 0.4 |  |  | 3 |  |  |  |  | 100 |  |  |  |
| *Lateolabrax japonicus* | 1 |  |  |  |  |  | 0.1 | 1 |  |  |  |  | 100 |  |  |  |
| *Anguilla japonica* | 2 |  |  |  |  | 0.2 |  | 1 |  |  |  |  | 100 |  |  |  |
| *Elopichthys bambusa* | 1 |  |  | 0.1 |  |  |  | 1 |  |  |  |  | 100 |  |  |  |
| **N-I** |  |  |  |  |  |  |  |  | Contributions of nekton and insect > 70.1 | | | | | | | |
| *Pelteobagrus vachelli* | 40 |  |  | 0.8 | 2.6 | 1.3 |  | 32 | 35.3 | 2.7 | 6.7 | 7.7 | 34.9 | 6.5 |  | 6.3 |
| *Channa asiatica* | 4 |  |  |  |  | 0.4 |  | 2 | 31.2 |  | 4.4 | 7.9 | 53.4 | 0.6 |  | 2.5 |
| *Mastacembelus armatus* | 71 |  | 1.4 | 1.0 | 4.1 | 0.7 |  | 46 | 33.9 |  | 2.5 | 1.5 | 55.4 | 3.5 |  | 3.2 |
| *Clarias gariepinus** | 7 |  |  |  |  | 0.5 | 0.2 | 15 | 4.7 |  | 13.0 | 7.5 | 61.0 | 4.3 |  | 9.5 |
| *Channa gachua* | 1 |  |  |  |  | 0.1 |  | 1 | 8.9 |  | 8.9 |  | 68.0 | 7.7 |  | 6.6 |
| *Clarias fuscus* | 18 |  |  | 1.6 | 0.7 | 0.2 |  | 13 | 23.6 | 0.5 | 0.4 | 2.7 | 71.1 | 0.8 |  | 0.8 |
| *Pelteobagrus fulvidraco* | 195 |  | 0.6 | 6.0 | 15.5 | 2.3 | 0.1 | 72 | 15.2 | 9.2 | 1.9 | 6.3 | 58.7 |  |  | 8.7 |
| **I** |  |  |  |  |  |  |  |  | Contribution of insect > 64.2 | | | | | | | |
| *Glyptothorax fokiensis* | 4 |  | 0.2 |  |  |  |  | 5 | 100 |  |  |  |  |  |  |  |
| *Pseudobagrus adiposalis* | 13 |  | 0.7 |  |  |  |  | 11 | 98.6 | 1.3 | 0.1 |  |  |  |  |  |
| *Mystus macropterus* | 2 |  |  |  | 0.3 |  |  | 2 | 87.1 | 5.1 | 1.1 |  | 6.7 |  |  |  |
| *Micronemacheilus pulcher* | 31 |  | 1.0 | 0.4 | 1.5 |  |  | 19 | 86.2 | 5.8 | 2.7 | 2.8 |  |  |  | 2.6 |
| *Schistura fasciolata* | 55 | 6.6 | 1.2 |  |  |  |  | 63 | 89.9 | 1.5 | 0.4 | 3.6 |  | 0.3 |  | 4.3 |
| *Rhinogobius giurinus* | 670 | 26.6 | 12.5 | 14.3 | 14.1 | 10.3 |  | 209 | 78.7 | 4.5 | 7.2 | 0.7 | 3.0 | 1.5 |  | 4.4 |
| *Rhinogobius duospilus* | 8 | 1.3 |  |  | 0.3 |  |  | 1 |  |  |  |  |  |  |  |  |
| *Rhinogobius brunneus* | 1 |  |  |  |  | 0.1 |  | 1 |  |  |  |  |  |  |  |  |
| *Leiocassis virgatus* | 22 |  | 0.2 |  | 2.3 |  |  | 12 | 79.5 | 2.3 | 12.5 | 0.6 |  |  | 0.1 | 5.1 |
| *Macropodus opercularis* | 11 |  | 0.6 |  |  | 0.1 |  | 11 | 82.4 | 3.4 | 10.2 |  |  | 0.1 | 0.3 | 3.6 |
| *Opsariichthys bidens* | 622 | 31.0 | 23.0 | 9.8 |  |  |  | 145 | 70.9 | 3.3 | 1.5 | 1.0 | 21.9 |  |  | 1.4 |
| *Acrossocheilus parallens* | 135 | 7.8 | 5.5 |  |  |  |  | 80 | 64.2 | 19.0 | 14.3 |  | 0.3 |  |  | 2.1 |
| *Gambusia affinis* | 2 |  | 0.1 |  |  |  |  | 4 | 78.4 | 17.7 |  |  |  | 3.9 |  |  |
| **I-D** |  |  |  |  |  |  |  |  | Contributions of insect and detritus > 60.6 | | | | | | | |
| *Monopterus albus* | 11 | 2.2 |  |  |  |  |  | 2 | 53.4 | 0.4 | 2.2 |  | 17.3 | 2.6 |  | 24.1 |
| *Botia robusta* | 1 |  |  |  | 0.2 |  |  | 1 | 54.3 | 9.6 | 5.3 | 5.5 |  |  |  | 25.2 |
| *Cobitis sinensis* | 5 |  |  | 0.5 | 0.3 |  |  | 5 | 56.6 | 9.3 | 2.1 | 4.8 |  |  | 0.8 | 26.3 |
| *Cobitis arenae* | 1 |  |  |  | 0.1 |  |  | 2 |  |  |  |  |  |  |  |  |
| *Misgurnus anguillicaudatus* | 51 | 0.1 | 1.8 | 1.2 | 0.7 | 0.6 |  | 68 | 32.7 | 17.3 | 6.9 | 13.4 |  | 0.4 | 1.3 | 28.0 |
| *Saurogobio dabryi* | 18 |  |  | 1.4 | 1.2 |  |  | 8 | 43.4 | 10.8 | 13.7 | 3.7 |  |  |  | 28.3 |
| **I-M** |  |  |  |  |  |  |  |  | Contributions of insect and mollusc > 69.1 | | | | | | | |
| *Gobiobotia meridionalis* | 11 |  |  |  | 1.4 |  |  | 1 | 34.6 | 11.8 | 34.6 | 7.7 |  | 2.0 | 1.1 | 8.2 |
| *Microphysogobio elongata* | 35 |  | 0.1 | 0.2 | 4.0 |  |  | 18 | 37.0 | 12.3 | 37.0 | 3.6 | 0.7 |  | 0.5 | 9.0 |
| *Acrossocheilus labiatus* | 143 | 0.9 | 6.8 | 3.0 |  |  |  | 41 | 49.7 | 5.9 | 28.5 | 11.6 |  |  | 0.0 | 4.2 |
| *Pseudogobio vaillanti* | 4 |  |  |  | 0.5 |  |  | 6 | 56.3 | 5.7 | 26.7 |  |  |  |  | 11.2 |
| *Hemibarbus medius* | 45 |  | 1.0 | 1.6 | 2.2 |  |  | 26 | 46.2 | 3.2 | 47.2 | 1.1 |  |  |  | 2.2 |
| *Microphysogobio kiatingensis* | 5 |  |  | 0.9 |  |  |  | 4 | 54.1 | 6.8 | 35.0 |  |  |  |  | 4.1 |
| *Sarcocheilichthys nigripinnis* | 96 |  | 0.4 | 7.6 | 5.1 |  |  | 31 | 49.6 | 8.6 | 37.3 | 0.6 |  |  |  | 3.9 |
| **E-I** |  |  |  |  |  |  |  |  | Contributions of epiphyte and insect > 68.5 | | | | | | | |
| *Squalidus argentatus* | 52 |  | 2.6 | 0.3 | 0.5 |  |  | 15 | 35.0 | 33.5 | 17.5 | 5.5 |  |  | 0.4 | 8.1 |
| *Squalidus wolterstorffi* | 2 |  |  |  | 0.3 |  |  | 3 |  |  |  |  |  |  |  |  |
| *Abbottina rivularis* | 25 |  | 0.1 |  | 2.9 |  |  | 7 | 46.7 | 31.5 | 10.5 | 2.9 |  |  | 0.1 | 8.3 |
| *Zacco platypus* | 462 | 14.5 | 17.5 | 13.0 |  |  |  | 106 | 49.1 | 34.2 | 2.5 | 6.3 | 3.4 | 0.6 |  | 3.9 |
| *Spinibarbus caldwelli* | 18 |  | 1.0 |  |  |  |  | 19 | 29.2 | 43.7 | 1.0 | 24.1 |  |  |  | 2.0 |
| *Carassioides cantonensis* | 15 |  |  |  |  | 1.3 |  | 11 | 34.7 | 48.1 |  | 6.5 |  |  | 2.8 | 7.8 |
| *Acheilognathus barbatulus* | 65 |  | 0.2 | 1.5 | 2.8 | 2.6 |  | 26 | 39.5 | 36.7 | 1.6 | 1.4 |  | 0.03 | 1.0 | 19.7 |
| *Puntius semifasciolatus* | 80 |  | 3.0 | 4.1 |  | 0.2 |  | 60 | 34.7 | 42.1 | 3.6 | 1.5 |  | 0.6 | 0.7 | 16.8 |
| *Rhodeus ocellatus* | 38 |  |  | 1.4 | 2.7 | 0.8 |  | 32 | 35.6 | 35.7 | 2.3 | 6.9 |  | 0.04 | 1.4 | 18.0 |
| *Rhodeus sinensis* | 162 |  | 3.5 | 4.0 | 4.8 | 3.5 |  | 43 |  |  |  |  |  |  |  |  |
| **E** |  |  |  |  |  |  |  |  | Contribution of epiphyte > 73.1 | | | | | | | |
| *Pseudogastromyzon changtingensis* | 16 | 3.1 |  |  |  |  |  | 26 | 12.2 | 73.2 | 0.3 |  |  |  |  | 14.3 |
| *Vanmanenia pingchowensis* | 71 | 4.5 | 2.7 |  |  |  |  | 21 | 14.2 | 76.2 | 1.3 | 1.1 |  | 0.2 |  | 7.0 |
| *Vanmanenia hainanensis* | 8 | 0.8 | 0.3 |  |  |  |  | 15 |  |  |  |  |  |  |  |  |
| *Vanmanenia gymnetrus* | 2 | 0.3 |  |  |  |  |  | 16 |  |  |  |  |  |  |  |  |
| **E-D** |  |  |  |  |  |  |  |  | Contributions of epiphyte and detritus > 52.7 | | | | | | | |
| *Carassius auratus* | 262 | 0.4 | 7.0 | 11.7 | 4.9 | 1.6 | 1.1 | 172 | 7.8 | 25.6 | 14.9 | 22.4 |  | 0.0 | 2.2 | 27.1 |
| *Osteochilus salsburyi* | 81 |  |  |  | 7.0 | 2.3 | 0.2 | 11 | 13.8 | 30.7 | 18.5 | 10.2 |  | 0.1 | 1.3 | 25.4 |
| *Acheilognathus chankaensis* | 32 |  |  |  |  | 2.3 | 0.9 | 5 | 4.1 | 40.1 | 3.1 | 12.4 |  | 0.5 | 9.6 | 30.1 |
| *Garra orientalis* | 6 |  |  |  |  | 0.5 |  | 1 | 9.3 | 49.9 | 6.2 | 4.3 |  | 1.5 | 1.7 | 27.1 |
| *Pseudorasbora parva* | 46 |  | 0.6 | 5.5 |  |  |  | 15 | 12.7 | 47.1 | 1.3 | 2.6 |  |  | 4.0 | 32.4 |
| **H** |  |  |  |  |  |  |  |  | Contribution of hydrophyte = 97.8 | | | | | | | |
| *Ctenopharyngodon idellus* | 21 |  |  | 0.2 | 1.5 | 0.8 |  | 18 | 0.2 | 0.7 | 1.3 | 97.8 |  |  |  |  |
| **H-D** |  |  |  |  |  |  |  |  | Contributions of hydrophyte and detritus > 61.0 | | | | | | | |
| *Pseudohemiculter dispar* | 42 |  |  |  | 0.2 | 3.2 | 0.5 | 21 | 12.0 | 8.5 | 7.7 | 30.7 |  | 1.9 | 5.9 | 33.4 |
| *Hemiculterella wui* | 1 |  |  |  |  |  | 0.2 | 1 |  | 17.1 |  | 38.9 |  |  | 13.4 | 30.5 |
| *Tilapia zillii** | 306 |  |  |  | 0.5 | 22.4 | 7.3 | 74 | 0.1 | 19.4 | 6.1 | 39.3 |  | 1.3 | 1.2 | 32.6 |
| **H-M** |  |  |  |  |  |  |  |  | Contributions of hydrophyte and mollusc > 53.0 | | | | | | | |
| *Sinibrama melrosei* | 12 |  |  |  | 1.6 |  |  | 3 | 28.0 | 5.4 | 12.6 | 40.5 |  |  |  | 13.5 |
| *Acrossocheilus beijiangensis* | 8 |  |  | 0.1 | 1.0 |  |  | 5 | 23.1 | 7.5 | 17.8 | 38.9 |  |  |  | 12.7 |
| *Rasborinus lineatus* | 2 |  |  |  |  | 0.2 |  | 1 | 19.5 | 4.8 | 22.5 | 39.1 |  |  | 1.2 | 12.9 |
| *Cyprinus flammans* | 9 |  |  | 0.2 |  | 0.7 |  | 27 | 10.4 | 4.6 | 37.5 | 37.6 |  |  |  | 9.8 |
| *Cyprinus carpio* | 146 |  | 0.9 | 3.2 | 4.7 | 4.1 | 3.9 | 68 | 2.2 | 2.6 | 50.8 | 31.0 |  | 0.7 | 2.6 | 10.1 |
| *Cyprinus carpio carpio* | 8 |  |  | 0.8 |  |  | 0.4 | 18 |  |  |  |  |  |  |  |  |
| **M** |  |  |  |  |  |  |  |  | Contribution of mollusc > 50.2 | | | | | | | |
| *Megalobrama terminalis* | 1 |  |  |  |  |  | 0.1 | 2 |  | 6.8 | 70.5 | 1.7 |  |  | 4.3 | 16.8 |
| *Parabramis pekinensis* | 117 |  |  |  |  | 1.8 | 14.4 | 55 |  | 5.8 | 61.0 | 3.9 |  | 2.0 | 1.0 | 26.3 |
| *Squaliobarbus curriculus* | 63 |  |  |  |  | 1.2 | 7.4 | 48 |  | 4.3 | 50.3 | 7.4 | 7.9 | 1.5 | 1.9 | 26.9 |
| **A-N-M** |  |  |  |  |  |  |  |  | Contributions of annelid, nekton, and mollusc > 53.9 | | | | | | | |
| *Glossogobius giuris* | 15 |  |  |  |  |  | 2.3 | 13 | 6.8 | 4.2 | 17.5 | 5.1 | 35.9 | 18.3 | 4.3 | 7.9 |
| *Mystus guttatu* | 3 |  |  |  |  | 0.3 |  | 2 | 9.8 |  | 21.4 | 6.5 | 37.4 | 16.5 |  | 8.4 |
| *Hypseleotris compressocephalus* | 1 |  |  |  |  | 0.1 |  | 1 | 13.0 | 8.1 | 23.7 | 7.0 |  | 35.0 |  | 13.1 |
| *Anabas testudineus* | 5 |  |  |  |  | 0.2 | 0.4 | 2 | 11.6 | 2.7 | 19.8 | 9.6 | 16.2 | 17.9 | 4.9 | 17.4 |
| *Eleotris oxycephala* | 4 |  |  |  |  |  | 0.7 | 2 | 3.2 |  | 30.8 | 10.8 | 20.0 | 22.9 |  | 12.4 |
| *Odontamblyopus rubicundus* | 17 |  |  |  |  |  | 2.6 | 6 | 8.2 |  | 14.6 | 14.7 | 24.7 | 26.2 | 1.4 | 10.2 |
| **D-E** |  |  |  |  |  |  |  |  | Contributions of detritus and epiphyte > 62.7 | | | | | | | |
| *Paramisgurnus dabryanus* | 7 |  |  | 0.1 |  | 0.5 |  | 5 | 4.8 | 12.1 | 14.1 | 11.2 |  | 0.4 | 6.7 | 50.6 |
| *Xenocypris argentea* | 18 |  |  | 0.5 | 1.2 | 0.4 |  | 15 | 0.8 | 30.4 | 3.2 | 12.3 |  | 0.2 | 4.9 | 48.2 |
| *Xenocypris davidi* | 1 |  |  | 0.1 |  |  |  | 3 |  |  |  |  |  |  |  |  |
| *Xenocypris microlepis* | 3 |  |  |  |  | 0.3 |  | 7 |  |  |  |  |  |  |  |  |
| *Hemiculter leucisculus* | 30 |  |  |  | 0.9 | 1.8 | 0.4 | 12 | 8.2 | 20.4 | 8.0 | 10.0 | 1.0 |  | 3.4 | 48.9 |
| *Oreochromis niloticus** | 11 |  |  | 0.4 | 1.1 |  |  | 26 | 0.8 | 22.8 | 8.8 | 17.4 |  |  | 2.0 | 48.3 |
| **D** |  |  |  |  |  |  |  |  | Contribution of detritus > 61.2 | | | | | | | |
| *Cirrhinus cirrhosus** | 241 |  |  |  | 0.3 | 6.5 | 24.7 | 40 | 0.2 | 15.3 | 2.4 | 1.6 |  | 0.3 | 6.2 | 73.9 |
| *Labeo rohita** | 9 |  |  |  |  |  | 1.4 | 2 |  | 9.0 | 2.3 | 2.1 |  | 3.1 | 5.2 | 78.2 |
| *Cirrhinus molitorella* | 197 |  |  |  | 0.2 | 6.1 | 18.8 | 89 | 0.2 | 26.2 | 1.2 | 2.8 |  | 0.1 | 8.2 | 61.3 |
| *Hypostomus plecostomus** | 50 |  |  |  |  | 2.5 | 3.3 | 45 |  | 20.2 | 0.4 | 7.3 |  | 0.4 | 2.1 | 69.6 |
| *Prochilodus scrofa** | 2 |  |  |  |  |  | 0.3 | 1 |  | 21.5 | 8.4 |  |  |  | 3.6 | 66.5 |
| **P-D** |  |  |  |  |  |  |  |  | Contributions of plankton and detritus > 80.8 | | | | | | | |
| *Aristichthys noblis* | 43 |  |  |  |  | 3.6 | 0.4 | 10 |  | 15.2 | 1.2 | 2.7 |  |  | 38.9 | 41.9 |
| *Hypophthalmichthys molitrix* | 60 |  |  |  |  | 4.8 | 0.9 | 14 |  | 12.4 |  | 2.8 |  |  | 47.8 | 37.0 |
| *Pseudolaubuca sinensis* | 14 |  |  |  |  | 1.2 |  | 1 | 6.2 | 8.3 |  |  |  |  | 50.8 | 34.7 |
| **P** |  |  |  |  |  |  |  |  | Contribution of plankton > 59.9 | | | | | | | |
| *Coilia grayii* | 84 |  |  |  |  | 4.3 | 5.3 | 24 | 3.9 |  |  |  | 12.7 |  | 76.7 | 6.7 |
| *Coilia mystus* | 5 |  |  |  |  |  | 0.8 | 8 |  |  |  |  |  |  |  |  |
| *Collichthys lucidus* | 2 |  |  |  |  |  | 0.3 | 10 | 1.9 |  |  |  | 22.0 |  | 60.0 | 16.1 |

**Table S3** Abundance (individual number per square meter, inds./m^2^) of macroinvertebrates and biomass (wet weight, g/m^2^) of hydrophytes and plant debris of the 6 sampling sites along the East River during the rainy and dry seasons (standard deviation in parentheses)

| Parameters |  | Site 1 | |  |  | Site 2 | |  |  | Site 3 | |  |  | Site 4 | |  |  | Site 5 | |  |  | Site 6 | |  |
| --- | --- | --- | --- | --- | --- | --- | --- | --- | --- | --- | --- | --- | --- | --- | --- | --- | --- | --- | --- | --- | --- | --- | --- | --- |
|  | Rainy | | Dry | | Rainy | | Dry | | Rainy | | Dry | | Rainy | | Dry | | Rainy | | Dry | | Rainy | | Dry | |
| Diptera (inds./m^2^) | 759  (283) | | 462  (201) | | 2066  (984) | | 1420  (432) | | 1.8  (0.7) | | 1.4  (1.6) | | 3.2  (3.0) | | 4.0  (3.1) | | <0.1 | | <0.1 | | <0.1 | | <0.1 | |
| Ephemeroptera (inds./m^2^) | 817  (203) | | 452  (156) | | 790  (279) | | 609  (294) | | 0.3  (0.2) | | 0.1  (0.2) | | 1.7  (1.5) | | 1.5  (1.2) | | 0.8  (0.6) | | - | | - | | - | |
| Coleoptera (inds./m^2^) | 79  (32) | | 72  (43) | | 960  (140) | | 1001  (97) | | 0.8  (0.4) | | 0.7  (0.4) | | 0.4  (0.5) | | - | | - | | - | | - | | - | |
| [Trichoptera](javascript:;) (inds./m^2^) | 270  (119) | | 470  (202) | | 857  (136) | | 468  (78) | | - | | - | | - | | - | | - | | - | | - | | - | |
| Lepidoptera (inds./m^2^) | 6.5  (4.1) | | 6.7  (5.4) | | 44  (21) | | 56  (17) | | - | | - | | - | | - | | - | | - | | - | | - | |
| Odonata (inds./m^2^) | 13  (12) | | 4.6  (5.5) | | 51  (39) | | 45  (27) | | 0.2  (0.1) | | 0.1  (0.2) | | 1.7  (0.7) | | 0.2  (0.3) | | 1.2  (0.5) | | 1.3  (1.2) | | <0.1 | | <0.1 | |
| Megaloptera (inds./m^2^) | 5.2  (2.1) | | 1.8  (1.6) | | 4.6  (2.3) | | 2.9  (1.8) | | - | | - | | - | | - | | - | | - | | - | | - | |
| Hemiptera (inds./m^2^) | <0.1 | | <0.1 | | 0.9  (0.4) | | 0.1  (0.2) | | 0.4  (0.1) | | 0.2  (0.1) | | <0.1 | | <0.1 | | - | | 0.3  (0.1) | | - | | - | |
| Decapoda (inds./m^2^) | - | | - | | 2.5  (1.7) | | 1.2  (0.8) | | 3.3  (1.7) | | 2.1  (1.1) | | 4.8  (2.1) | | 3.9  (2.4) | | 10  (3.1) | | 6.7  (2.5) | | 19  (5.3) | | 13  (2.8) | |
| Hirudinea (inds./m^2^) | 16  (5.9) | | 20  (3.1) | | 43  (11) | | 22  (7.1) | | 0.5  (0.4) | | - | | - | | - | | - | | - | | - | | - | |
| Bivalvia (inds./m^2^) | 10  (3.2) | | 5.2  (2.1) | | 53  (20) | | 36  (11) | | 21  (12) | | 14  (6.0) | | 11  (8.1) | | 8.9  (4.2) | | 39  (12) | | 24  (9.2) | | 67  (21) | | 41  (12) | |
| Gastropoda (inds./m^2^) | 32  (6.2) | | 21  (5.6) | | 50  (21) | | 48  (14) | | 6.9  (1.1) | | 2.8  (0.2) | | 2.3  (0.4) | | 1.1  (0.1) | | 3.2  (2.2) | | 2.4  (1.0) | | 5.4  (2.1) | | 4.8  (1.4) | |
| Oligochaeta (inds./m^2^) | 30  (34) | | 3.7  (5.2) | | 33  (16) | | 13  (6.1) | | 4.0  (0.9) | | - | | 10  (2.2) | | - | | 43  (22) | | 27  (12) | | 58  (12) | | 352  (126) | |
| Polychaete (inds./m^2^) | - | | - | | - | | - | | - | | - | | - | | - | | - | | - | | 15  (18) | | 14  (11) | |
| Submerged plants (g/m^2^) | 5.7  (4.1) | | 8.2  (4.1) | | 28  (14) | | 41  (21) | | 2.4  (2.1) | | 1.1  (0.5) | | 0.4  (0.6) | | 0.3  (0.4) | | - | | - | | - | | - | |
| Plant debris (g/m^2^) | 13  (6.5) | | 12  (4.5) | | 44  (31) | | 15  (7.0) | | 12  (10) | | 1.5  (0.7) | | 1.6  (0.4) | | 1.0  (0.3) | | 0.3  (0.5) | | 0.1  (0.1) | | 0.2  (0.1) | | 0.1  (0.2) | |

**FIGURE S1** Seasonal differences in the distribution and composition of fish trophic guilds in terms of abundance

**FIGURE S2** Seasonal differences in the distribution and composition of fish trophic guilds in terms of biomass

**FIGURE S3** Fish indicator species of trophic links and their utilised prey items along the East River


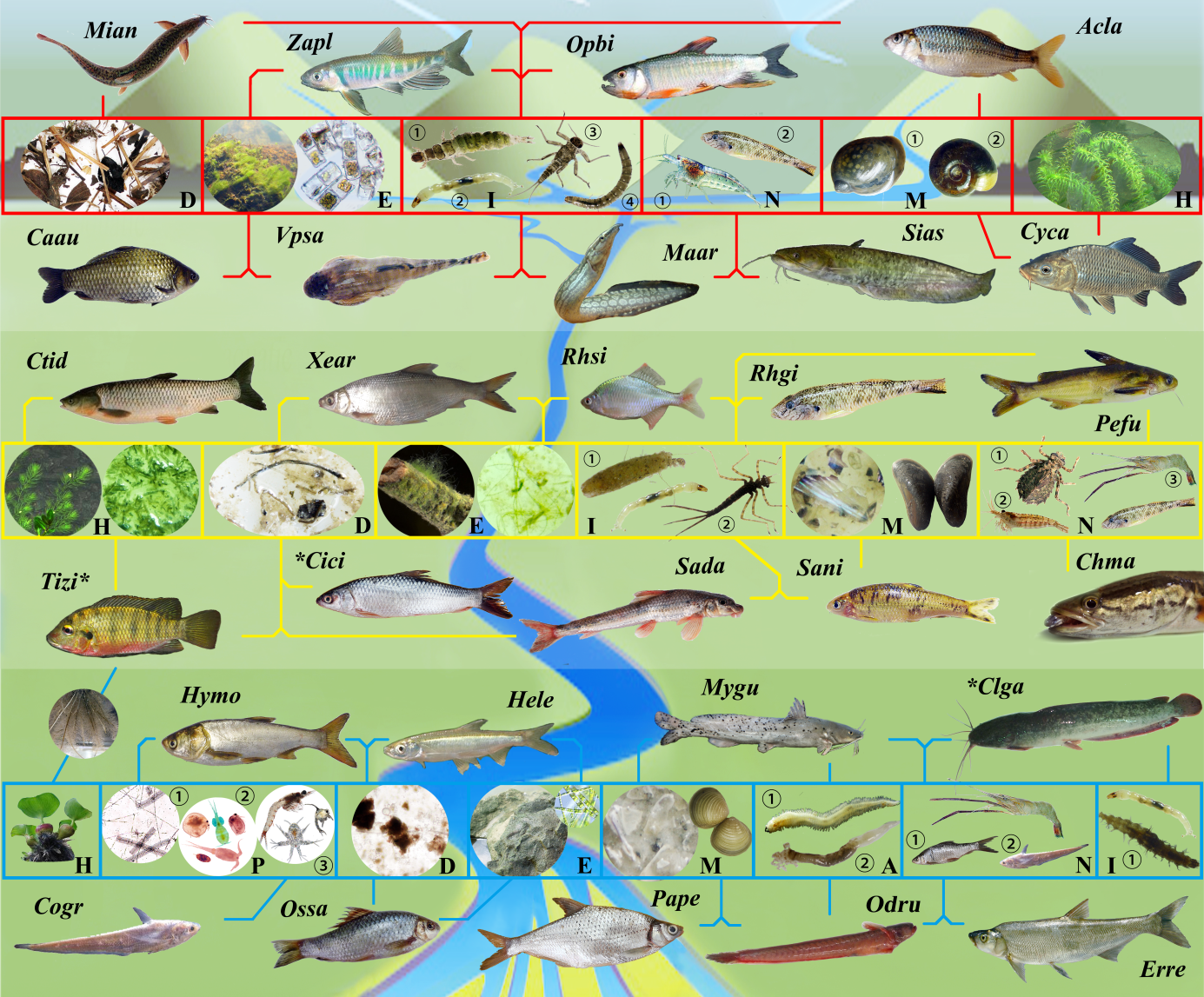
The site-specific prey items in separate rectangle grids are connected to the corresponding fish indicator species, with upstream trophic links in red (the upper at site 1, the lower at site 2), midstream in yellow (the upper at site 3, the lower at site 4), and downstream in blue (the upper at site 5, the lower at site 6). Fish indicators that feed on more than one major prey item are connected with 2-3 link ends, and the direction of their mouth points to the prey item that contributes most to the diet composition of individual species.

In a longitudinal gradient, the prey items utilised by fish indicator species are as follows:

1) Upstream prey in red grids: detritus (D, coarse debris of aquatic or terrestrial plants), epiphytes (E, Melosiraceae of epilithic diatoms), insects (I, including Polycentropodidae ①, Chironomidae ②, Heptageniidae ③, and Heteroceridae ④), nekton (N, including shrimp *Neocaridina denticulate* and fish *Rhinogobius giurinus*), molluscs (M, including soft-shelled *Radix* ① and *Hippeutis* ② of gastropods), hydrophytes (H, submerged *Hydrilla verticillata*);

2) Midstream prey in yellow grids: hydrophytes (H, submerged *Myriophyllum verticillatum*), detritus (D, rotten vegetal debris and suspended particulate matter), epiphytes (E, Cladophoraceae of filamentous green alga), insects (I, including Hydroptilidae ①, Protoneuridae ②, and Chironomidae), molluscs (M, *Limnoperna lacustris* of bivalves), nekton (N, including larvae of odonate Gomphidae ①, *Caridina nilotica* ② and *Macrobrachium nipponense* ③ of shrimps);

3) Downstream prey in blue grids: hydrophytes (H, the roots of floating *Eichhornia crassipes*), plankton (P, including phytoplankton ①, copepods and cladocerans ②, and pelagic decapod larvae ③), detritus (D, sediment organic matter), molluscs (larvae of bivalve *Corbicula fluminea*), annelids (A, including Nereidae or Nephtyidae ① and Sabellidae ② of polychaetes), nekton (N, including shrimp *M*. *nipponense*, juvenile fish of *Cirrhinus molitorella* ① and *Coilia grayii* ②), insects (I, including Nymphulinae ① and Chironomidae).

**References**

Barbour, M. T., J. Gerritsen, B. Snyder & J. Stribling. (1999). Rapid bioassessment protocols for use in streams and wadeable rivers. USEPA, Washington.

Hauer, F. R. & G. A. Lamberti. (2007). Methods in Stream Ecology. Academic Press, London.

SEPA. (2002). Standard Methods for Water and Wastewater Monitoring and Analysis. China Environmental Science Press, Beijing.

Wang, Z. Y., J. H. W. Lee, D. S. Cheng & X. H. Duan. (2008). Benthic invertebrates investigation in the East River and habitat restoration strategies. *Journal of Hydro-Environment Research*, **2**, 19-27. doi:10.1016/j.jher.2008.05.005.
